# A Framework for Short-Range Wireless Power and Data Transfer in Miniaturized High-Power, High-Bandwidth Implants

**DOI:** 10.3390/mi17080989

**Published:** 2026-08-21

**Authors:** Lyssa Ramaut, Pieterjan Polfliet, Gilles Callebaut, Liesbet Van der Perre

**Affiliations:** 1 Department of Electrical Engineering, Katholieke Universiteit Leuven (KU Leuven), 9000 Ghent, Belgium; 2 Cochlear Technology Centre Belgium, Cochlear Limited, 2800 Mechelen, Belgium

**Keywords:** miniaturized high-performance implants, wireless power transfer, wireless data transfer, power transfer efficiency, high data rate, design guidelines

## Abstract

As implantable medical devices become increasingly miniaturized while demanding higher power levels, longer lifetimes, and larger data throughput, conventional powering and communication approaches are reaching their practical limits. Consequently, wireless power and data transfer have emerged as key enabling technologies for next-generation implantable systems. However, designing wireless links that simultaneously satisfy these requirements while remaining compact, efficient, and safe remains a significant challenge. To address this, this paper introduces an exploration and evaluation framework for selecting and co-designing short-range wireless power and data transfer technologies in medical devices such as cochlear and retinal implants. Guided by application requirements and relevant safety standards, the framework evaluates candidate technologies based on their operating principles, performance, and integration complexity. Based on this analysis, resonant inductive coupling is identified as the preferred approach for wireless power transfer, while both coil-based and antenna-based solutions are considered for wireless data transfer. Additionally, the paper compares architectures for integrated wireless power and data transfer, including both single- and multiple-link designs, and reviews strategies for uplink communication. The framework aims to guide the development of future sensory neuroprostheses that are smaller, safer, and capable of higher performance through optimized wireless link design.

## 1. Introduction

Implantable medical technologies are rapidly evolving beyond their traditional applications, driven by ongoing research into novel therapies for a growing range of neurological, sensory, and systemic pathologies. In addition to established devices such as cochlear and cardiac implants, significant research is focused on emerging implantable systems for vision restoration, brain-to-machine interfaces, and neuromodulation (machine-to-brain). At the same time, substantial effort is devoted to enhance existing implant technologies by increasing functionality while further reducing implant size. The latter can simplify the surgical procedure and make the overall system less visible from the outside. Miniaturizing the implant therefore addresses two major barriers: aesthetic concerns and surgical complexity [1], that candidates may face today to decide to go for treatment.

Traditionally, implants rely on internal batteries to supply energy. While effective, batteries fundamentally limit implant lifetime, particularly in applications with higher power demands. Moreover, since battery energy capacity scales with physical size, high-performance implants require relatively large batteries, which conflicts with the strict volumetric constraints imposed by implantation and anatomical considerations. As a result, Wireless Power Transfer (WPT) provides an attractive alternative, as the energy source can be placed externally, enabling higher available energy capacity while reducing implant size and eliminating the need for battery replacement. Many implants also rely on a rechargeable battery, which itself involves a wireless power link to recharge the battery and a wireless data link for the feedback mechanism that maintains the constant-current (CC) and constant-voltage (CV) charging states [2]. In parallel, many applications also require Wireless Data Transfer (WDT) from an external unit to the implant, with data rates ranging from hundreds of kilobits per second to several megabits per second, to transmit stimulation-related information such as audio or video data. In addition, feedback from the implant to the external unit, such as neural recordings or device status information, is often required, necessitating reliable bidirectional wireless communication through biological tissue.

This paper focuses on miniaturized implantable systems with short transmission distances, power requirements on the order of tens of milliwatts, and data rate requirements ranging from hundreds of kilobits per second to several megabits per second. It specifically addresses the wireless power and data transfer between the external transmitter and the implanted receiver, and not the power management between an on-implant charger and its battery, which is therefore out of scope. Cochlear and retinal implants are both representative applications that pose these requirements. Cochlear implants typically require wireless power levels in the order of 10 mW to 100 mW while supporting data rates around 1 Mbps for audio and control information exchange [3]. Retinal implants require similar power requirements but impose even higher data rate demands, with reported values ranging from 2 Mbps to 20 Mbps due to high spatial resolution requirements [3]. The transmitter (TX)–receiver (RX) separation in these systems is relatively small. For cochlear implants, this distance typically ranges from only a millimeter up to approximately 12 mm, as the external transmitter and implanted receiver coil are separated mainly by skin tissue. For retinal implants, the separation is larger, on the order of 25 mm, since the transmitter is typically integrated into eyewear (e.g., glasses) and is therefore positioned at a greater distance from the implanted receiver [4]. Neural implants on the other hand also require extreme miniaturization, however they generally operate at lower power levels [5] (a few µW’s) and (sometimes no) data rates. In addition, their transmitter–receiver separation is also typically larger. Although these applications are not the primary focus of this work, several design insights and evaluation principles developed for cochlear and retinal implant systems may nevertheless be transferable.

Several review papers have addressed specific aspects of wireless technologies for implantable medical devices. In the area of wireless power transfer, existing reviews have compared the available power transfer mechanisms for implantable systems [6], examined wireless powering solutions for neural implants [5], and provided overviews of power transfer architectures, circuits, standards, and applications [7]. Other works have focused on implantable antennas, antenna miniaturization techniques, and RF communication links for implantable devices [8,9,10]. In parallel, recent reviews have specifically examined wireless power transfer and wireless powering strategies based on implantable antennas and deep-body bioelectronic receivers [11]. Furthermore, integrated wireless power and data transfer architectures and simultaneous wireless power and data transfer have been reviewed in [12,13,14].

Despite these contributions, literature still lacks a comprehensive review focused on miniaturized high-power, high-bandwidth implants. To address this gap, this paper provides an overview and exploration framework for the selection and co-design of the wireless power and data links for such implants. Guided by the requirements of cochlear and retinal implants and relevant safety constraints, candidate technologies are evaluated with respect to their operating principles, efficiency, bandwidth, robustness, size, and integration complexity. Based on this evaluation, the focus is narrowed to resonant inductive coupling for WPT, while both coil-based and antenna-based solutions are considered for WDT.

**Figure 1:**
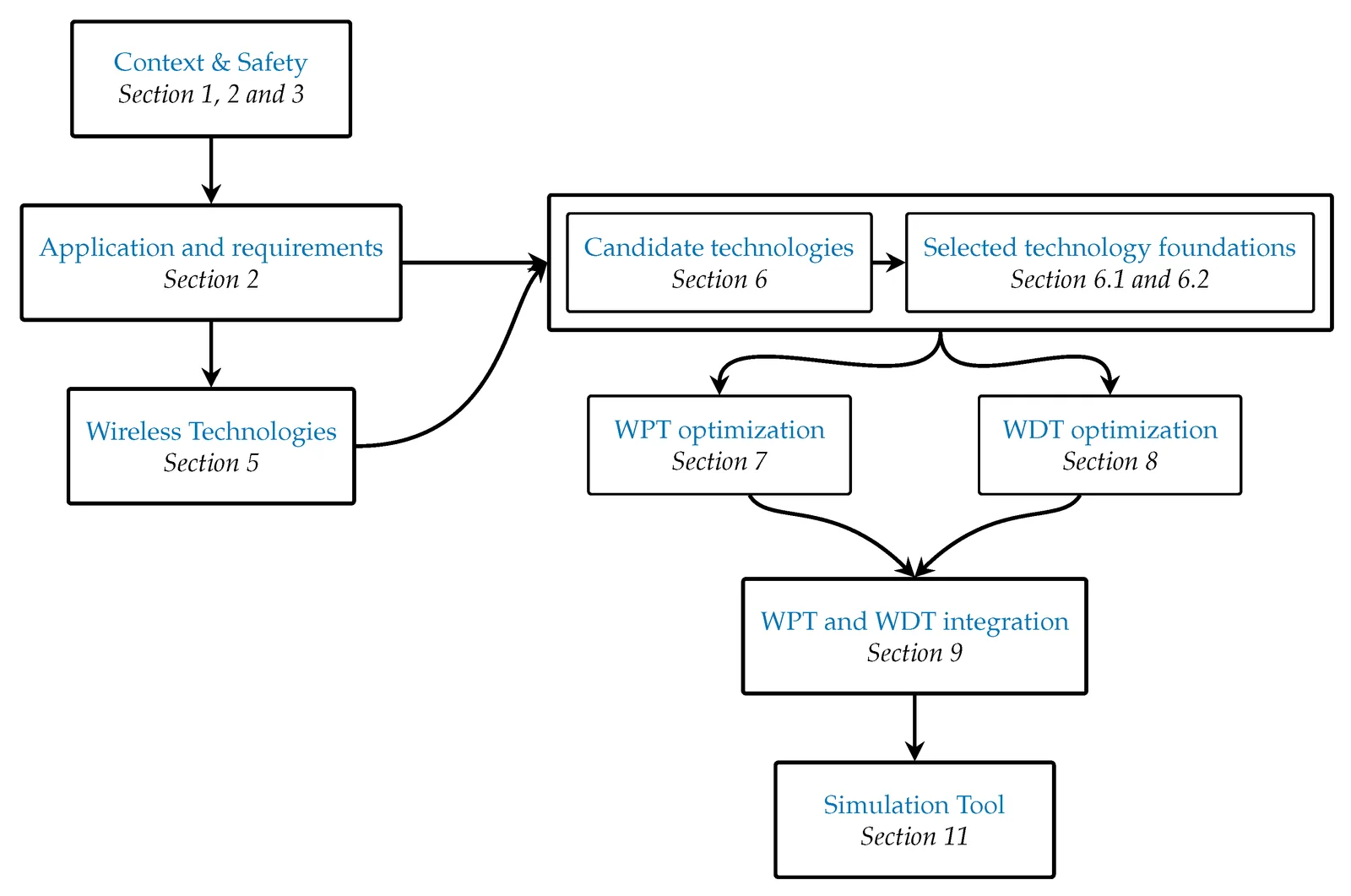
Schematic overview of this paper.

The remainder of this paper is organized as follows. Section 2 reviews the working principles of cochlear and retinal implants in more detail, while Section 3 presents the regulatory and safety requirements relevant to wireless power and data transfer in implantable medical devices, including exposure limits and thermal constraints. Section 4 defines the key application-level metrics that form the basis for the systematic evaluation in Section 5, where different wireless power and data transfer technologies are compared. As already indicated, this analysis motivates the selection of resonant coupling in Section 6 as the most suitable approach for miniaturized high-power implants, while identifying both coil- and antenna-based solutions for data transfer. Further, it also outlines the relevant operating frequency bands. Building on this, Section 7 focuses on WPT and its efficiency optimization strategies, whereas Section 8 evaluates WDT, considering trade-offs in terms of bandwidth (BW), safety, and spatial constraints. Subsequently, Section 9 addresses the integration of wireless power and data transfer, considering both single- and multi-link architectures, while Section 10 focuses on uplink data communication schemes. Finally, Section 11 outlines commonly used electromagnetic (EM) simulation tools, and Section 12 concludes the paper and highlights key design insights and future research directions. A schematic overview of this work is given in Figure 1.

## 2. Target Implant Applications

### 2.1. Cochlear Implants

**Figure 2:**
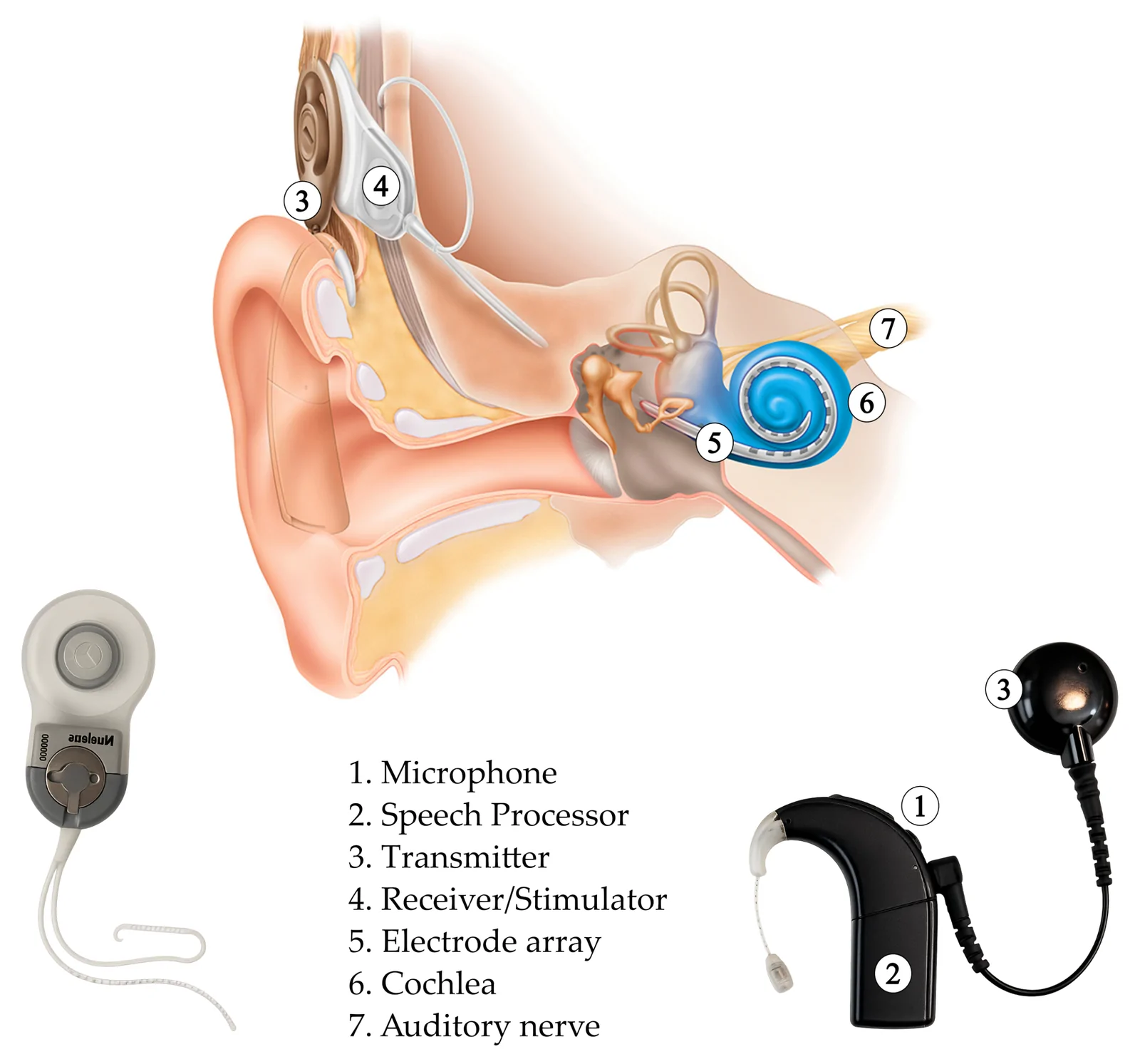
Schematic overview of a cochlear implant [19,20,21].

Cochlear Implant (CI) systems, as illustrated in Figure 2, have revolutionized the treatment of severe and profound hearing loss through electric stimulation of the auditory nerve [15]. The current generation of CI systems [16] is semi-implantable, featuring both an external and an internal component. The external unit captures sound and processes the data, while the implant incorporates a receiver that receives this data and translates it into neural stimulation pulses. Energy and data are both transferred from the external processor to this implanted receiver transcutaneous (through the skin) over an inductive link operating at 5 MHz. Today’s solutions use two closely coupled flat coils, one placed externally (transmitter), and one surgically implanted below the skin (receiver), separated by a distance ranging typically between 1 mm to 12 mm [17]. These coils are kept in place by magnets, making them very well aligned to ensure the highest magnetic coupling. The audio signal is translated into electrical pulses, which are routed by the receiver over the electrode lead to the cochlea (see Figure 2). More precisely, in the cochlea, an electrode array of up to 22 electrodes is implanted. Each electrode of this array is connected via an individual wire to the electrode lead [18].

The coil of the current cochlear implant takes up roughly half of the total implant footprint, as shown in Figure 2. By revisiting the transcutaneous inductive link, for example through a reduction of the coil diameter, the overall size of both the external transmitter and the implanted receiver can be reduced. This improves aesthetics and enables alternative implantation approaches that might eliminate the need to access the facial recess, thereby reducing surgical risks and improving patient outcomes [22].

### 2.2. Retinal Implants

**Figure 3:**
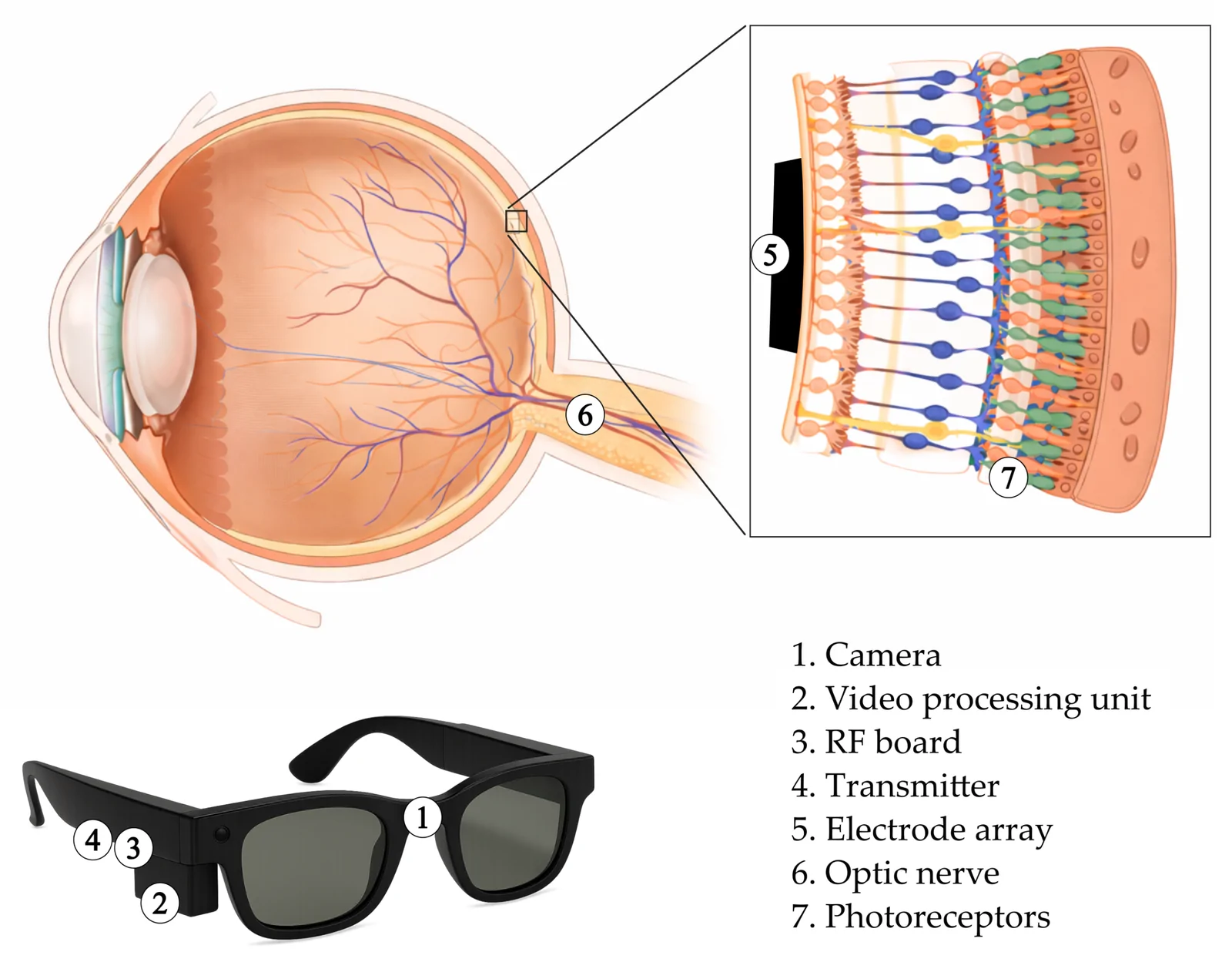
Schematic overview of a retinal implant [24,25].

Retinal implants, as shown in Figure 3, are neuroprosthetic devices designed to restore a limited form of visual perception in patients suffering from severe photoreceptor degeneration, such as retinitis pigmentosa or age-related retinal dystrophies [23]. In these conditions, the photoreceptors (rods and cones) are largely nonfunctional, while a significant portion of the inner retinal neurons and the optic nerve remain intact. Retinal prostheses aim to bypass the damaged photoreceptors by directly stimulating the surviving retinal neurons with electrical signals, thereby eliciting visual percepts in the brain.

The working principle is analogous to other sensory neuroprostheses, such as earlier described CIs. Visual information from the environment is first captured by an external imaging unit, typically a miniature camera mounted on a pair of glasses [26]. These images are processed by an external processing unit, where they are converted into stimulation patterns. Power and data are transferred from the external unit to the implant via a transcutaneous wireless link, using coils or antennas placed on either side of the skin. The implanted electronics decode the received data and generate controlled electrical stimulation pulses, which are delivered to the remaining retinal neurons.

In comparison to cochlear implants, retinal implants are subject to even more stringent spatial constraints, as the implanted electronics must be accommodated within or near the eye. Miniaturization of the wireless power and data link is therefore a key enabler for making retinal implants feasible.

## 3. Safety Requirements

The design and operation of wireless power and data transfer systems for implantable medical devices are governed by a combination of regulatory frameworks and technical safety standards, which together ensure patient safety, Electromagnetic Compatibility (EMC), and legal market access.

### 3.1. Regulatory Frameworks

In the United States, implantable medical devices are regulated by the Food and Drug Administration (FDA), which evaluates device safety and efficacy, while the Federal Communications Commission (FCC) governs the use of radio frequency (RF) emissions and spectrum compliance. In the European Union, similar frameworks apply. Medical implants must meet the requirements of the Medical Device Regulation (MDR) to obtain CE marking, which ensures essential safety and performance requirements throughout the device lifecycle, including compliance with applicable EMC standards. When the device incorporates wireless functionality, it must also comply with the Radio Equipment Directive (RED), which specifies additional requirements related to EMC, user safety and efficient use of the radio spectrum. Other regions apply similar dual regulatory structures, with separate authorities responsible for medical device approval and radio/spectrum compliance. An overview of the relevant regulatory bodies across all major regions is provided in Table 1.

### 3.2. Technical Standards

Compliance with the above regulatory frameworks is typically achieved by meeting the requirements defined in applicable technical standards. Many of these standards for implantable medical devices are developed by the International Organization for Standardization (ISO). Key examples include ISO 13485 [42], the international standard for quality management systems (QMS) specific to the medical device industry, ISO 14971 [43] for risk management, ISO 10993 [44] for biological evaluation and biocompatibility, and ISO 14708 [45,46], which specifies general safety requirements for active implantable medical devices, with dedicated parts for specific applications such as cochlear implants.

Within Europe, these international ISO standards are complemented and transposed into harmonized European standards, which can be applied to demonstrate conformity with applicable regulatory frameworks, such as the Medical Device Regulation (MDR) and the Radio Equipment Directive (RED). These harmonized standards are developed by European standardization bodies, including the European Telecommunications Standards Institute (ETSI).

For active implantable devices, for example, the European standard EN 45502-1 [47] represents the European implementation of ISO 14708 and specifies the essential safety and performance requirements for such devices. For wireless devices, on the other hand, ETSI also defines how to get compliance with the RED for specific frequency bands and technologies by using standardized measurement methods. A widely applied standard in this context is the ETSI EN 300 330 [48], which, among other requirements, specifies limits on radiated field strength for short-range inductive systems operating in the frequency range from 9 kHz to 30 MHz [49]. Other countries similarly rely on international standards like ISO to form the basis for their own regional standards.

### 3.3. Relevant Exposure Guidelines and Safety Limits

There are internationally recognized exposure guidelines that limit electromagnetic and thermal loading in biological tissue, with which wireless medical devices are required to comply. A widely adopted framework is provided by the International Commission on Non-Ionizing Radiation Protection (ICNIRP) [50], which specifies exposure limits for EM fields from 0 Hz to 300 GHz. The European Union, Australia, New Zealand, China and many Asian countries base their legally enforceable exposure limits on ICNIRP recommendations. Other regions, including the United States and Canada, rely on IEEE C95.1 [51] and Health Canada Safety Code 6 [52], respectively. Although these standards differ in specific details, they are based on the same underlying scientific principles and remain closely aligned with ICNIRP.

Each of these guidelines defines limits on EM exposure, including constraints on internal electric fields, the specific absorption rate (SAR), external electric and magnetic field strengths, and the permissible increase in tissue temperature, all of which are discussed in more detail below.

#### 3.3.1. Internal and External Electric and Magnetic Field Limits

ICNIRP, IEEE C95.1, and Health Canada Safety Code 6 define frequency-dependent basic restrictions on the internal root-mean-square (RMS) electric field in biological tissue to prevent nerve and muscle stimulation at low frequencies and excessive tissue heating at higher frequencies. In addition, corresponding external reference levels are specified for electric and magnetic field strength. The limits differ for public (uncontrolled) and occupational (controlled) exposure environments.

#### 3.3.2. Specific Absorption Rate (SAR) [53,54,55,56]

SAR quantifies the rate at which the electric-field is absorbed by biological tissue and is expressed in watts per kilogram (W/kg). The SAR is defined as

(1) $$\mathrm{SAR}=\frac{{\sigma |E|}^{2}}{\rho }$$

where σ denotes the electrical conductivity of the tissue, |E| is the root-mean-square electric field strength, and ρ is the tissue mass density. SAR is a primary metric to assess RF exposure and limit excessive tissue heating, particularly at higher frequencies. Even for implants using magnetic resonant coupling, SAR must be evaluated because time-varying magnetic fields induce secondary electric fields.

Regulatory limits on localized SAR depend on the exposure category (occupational or continuous), the exposed body region (whole-body, partial body or hands, wrists, feet & ankle), and the geographical region. For cochlear and retinal implants, which involve continuous stimulation in the head region (partial body), the maximum permissible SAR is 1.6 W/kg averaged over 1 g of tissue in the United States, Australia and New-Zealand, and 2 W/kg averaged over 10 g of tissue in the European Union, Japan and South-Korea.

#### 3.3.3. Maximum Permissible Exposure (MPE) [54,57]

MPE specifies the maximum allowable electric-field strength (V/m), magnetic-field strength (A/m), and power density (W/m^2^ or mW/cm^2^) to which humans may be exposed. These limits depend on the exposure category (occupational or continuous) and the operating frequency. For operation in the 2.4 GHz industrial, scientific and medical (ISM) band, the limit corresponds to a maximum power density of 10 W/m^2^ (equivalently 1 mW/cm^2^) for cochlear and retinal implants.

#### 3.3.4. Electromagnetic Compatibility (EMC) and Electromagnetic Interference (EMI) [48,58]

EMC and EMI requirements ensure that the implant neither causes nor is susceptible to unacceptable Electromagnetic Interference (EMI), which is critical for safe coexistence with other medical devices and electronic systems. Standards such as the IEC 55011 [58] (also known as CISPR 11, a subcommittee of the IEC) define emission limits and measurement methods for ISM equipment, including medical implants. The European version of this standard is EN 55011.

#### 3.3.5. Thermal Limits (CEM43 °C) [59]

The cumulative equivalent minutes at 43 °C (CEM43 °C) quantifies the biological effect of tissue heating over time. CEM43 °C represents the number of minutes for which tissue may be exposed to a given temperature profile that would result in the same biological effect as exposure at a constant external tissue temperature of 43 °C, the threshold at which tissue damage occurs. The calculated CEM43 °C is then compared to a thermal dose limit, set by standards, to ensure tissue temperatures remain within safe levels.

#### 3.3.6. Focalized Temperature Rise [53,54,60]

In addition to cumulative thermal dose limits, standards also restrict the allowable internal temperature increase in tissue surrounding the implant. The localized tissue temperature rise is typically limited to approximately 2 °C above normal body temperature, corresponding to a maximum tissue temperature of about 39 °C, to prevent thermal damage.

## 4. Application Requirements

Miniaturized implantable medical devices, such as sensory neuroprostheses including cochlear and retinal implants, require a substantial amount of power to effectively stimulate neural tissue at high stimulation rates, which is necessary to maintain signal fidelity and enable meaningful sensory restoration. In addition to power delivery, medical implants frequently require a reliable wireless data link for configuration, monitoring, and the transmission of stimulation or sensory information. While some applications rely only on low-rate telemetry, sensory implants such as cochlear and retinal prostheses demand continuous, high-rate data transmission, placing strong requirements on available bandwidth and link reliability.

Taken together, these considerations define a set of application-driven requirements that must be satisfied simultaneously by the wireless power and data links, enabling a systematic comparison across technologies. These requirements are listed below:

- Power Transfer Efficiency (PTE): Defined as the ratio of power delivered to the implant to the total transmitted power. A high Power Transfer Efficiency (PTE) lowers the required transmit power by reducing dissipative losses in the transmission link, thereby improving the utilization of the available power budget, which is critical in battery-powered systems. Moreover, improved efficiency is directly associated with reduced tissue heating, thereby facilitating compliance with the regulatory safety constraints outlined in Section 3.
- Bandwidth (BW): The usable bandwidth of the wireless link, which directly limits the achievable data rate. High-performance sensory implants, such as cochlear and retinal prostheses, require bandwidths sufficient to support data rates ranging from hundreds of kilobits per second to tens of megabits per second, depending on the application.
- Misalignment robustness: The sensitivity of the wireless link to lateral and angular displacement arising from anatomical variability between patients, surgical placement tolerances, and possible movement during daily activity. High robustness is required to ensure stable power delivery and reliable data communication without the need for realignment.
- Transmission distance: The maximum separation over which reliable operation can be maintained between the external transmitter and the implanted receiver. Relevant distances range from a few millimeters in cochlear implants to several centimeters in retinal implants, directly constraining both feasible link architectures and operating frequencies.
- Physical size: The physical dimensions of coils, antennas, matching networks, and associated electronics. Wireless link components often dominate the implant footprint and therefore constitute a primary bottleneck for further miniaturization.

## 5. Wireless Technologies

The design of wireless links for high-power, high-bandwidth miniaturized implants requires careful consideration of available technologies and their fundamental trade-offs. Both Wireless Power Transfer (WPT) and Wireless Data Transfer (WDT) are considered, as many implantable systems rely on a combination of the two. However, these functionalities impose partially conflicting requirements on the wireless link: efficient power transfer favors strong coupling and operation at relatively low frequencies to limit tissue losses, whereas data transmission benefits from wider bandwidths, typically associated with higher carrier frequencies.

As a starting point for selecting the most suitable technology, this section provides an overview of the main wireless approaches, including inductive, resonant, RF, optical, acoustic, and capacitive techniques. As a first point of comparison, Table 2 summarizes representative tissue attenuation values reported in the literature for these techniques, thereby offering insight into their fundamental propagation characteristics. It shows that approaches based on electromagnetic waves at optical frequencies experience severe attenuation due to strong absorption and scattering, whereas magnetically coupled techniques operate in the near-field regime and therefore exhibit negligible propagation losses. In contrast, RF, acoustic, and capacitive approaches exhibit intermediate attenuation levels, which are governed by the interaction of the propagating fields with biological tissue.

While these propagation characteristics provide an important first-order indication of feasibility, they do not, in isolation, determine overall system performance. Table 2 therefore also includes representative receiver dimensions required to achieve useful PTE and indicative data rate capabilities reported for implantable applications. Together, these metrics highlight important technology-dependent trade-offs, such as the possibility of highly miniaturized receivers at the expense of power-transfer performance, or higher data rates at the cost of increased tissue attenuation. However, technology selection ultimately depends on additional factors, including achievable link efficiency, transmitter dimensions, alignment sensitivity, regulatory safety limits, and application-specific requirements. The following subsections therefore present a detailed comparative analysis of these technologies, considering both their fundamental propagation behavior and their system-level implications.

### 5.1. Inductive Coupling and Magnetic Resonance

Inductive Wireless Power Transfer is particularly well suited for short-range power transfer as it operates in the near-field, where energy is transferred predominantly via magnetic fields [6]. Only secondary electric fields are present, which are induced by the time-varying magnetic fields. Although these electric fields are generally weak due to the low operating frequencies, typically in the range of a few megahertz, they can still impose exposure-related design constraints in implantable applications and must be carefully considered with respect to specific absorption rate (SAR) and regulatory limits [65]. Overall, their interaction with biological tissue remains significantly more limited compared to other technologies, such as radiative approaches (as illustrated in Table 2). However, the efficiency of inductive power transfer strongly depends on the coupling coefficient between the transmitter and receiver coils. This coefficient is highly sensitive to coil misalignment, angular orientation, and transmission distance, which poses significant challenges in implantable applications where relative movement between the external transmitter and the implanted receiver, such as in retinal implants, can occur.

To improve efficiency, resonant inductive coupling is often employed. In this approach, both the transmitter and receiver coils are combined with reactive elements, typically capacitors, to form LC resonant tanks tuned to the same resonant frequency $f_{c}$ [7]:

(2) $$f_{c}=\frac{1}{2\pi \sqrt{LC}}$$

where *L* is the coil inductance and *C* is the capacitance of the tank. At resonance, the reactive impedances of the inductor and capacitor cancel each other out ($Z_{l}$ = $-Z_{C}$), so the impedance of the *LC* circuit is only dominated by its resistive component. This process is commonly referred to as compensation, while the capacitors used to achieve it are referred to as compensation elements. Different ways of connecting these compensation elements to the transmitter and receiver coils give rise to different compensation topologies, which are discussed in Section 7.2. A well-designed resonant *LC* tank therefore exhibits a low effective resistance and, consequently, a high quality factor and PTE (as discussed in Section 6.1). This reflects minimal dissipative losses, meaning that energy is stored efficiently within the resonator. As a result, efficient power transfer is predominantly limited to short distances, where a receiving *LC* tank with a similarly high *Q*-factor can effectively capture and store the transmitted energy, even under moderate misalignment conditions. In such cases, both resonant tanks must be well matched in frequency and quality factor to enable efficient coupling. These capacitors act as compensation networks and can be connecting these compensation elements result in different compensation topologies, which are discussed in Section 7.2.

In addition to power transfer, inductive and resonant links can also support short-range wireless data transmission. In such systems, data transfer is typically achieved by modulating the amplitude, frequency, or phase of the carrier signal (e.g., load-shift keying or frequency-shift keying), allowing bidirectional communication over the same magnetic link. Although the achievable data rates are limited compared to far-field RF communication, they are generally sufficient for low-to-moderate bandwidth applications.

### 5.2. RF Coupling

In contrast to inductive coupling, RF-based WPT uses EM waves and can function in both the near and far-field. One major advantage of RF WPT is its reduced sensitivity to alignment of the transmitter and receiver, allowing power delivery over longer distances compared to inductive systems [6].

However, as shown in Table 2, RF waves are strongly attenuated in biological tissue, primarily due to dielectric losses associated with their electric fields. For example, at 2.45 GHz, the attenuation ranges approximately from 0.7 dB/cm to 0.8 dB/cm in adipose tissue up to about 6.9 dB/cm in the small-intestine tissue, depending on the dielectric properties of the tissue [62,66,67,68]. This strong attenuation in the small intestines originates from the high relative permittivity ($\epsilon_{r}\approx 54.4$) and conductivity (σ≈3.17 S/m) of biological tissues at microwave frequencies [9,62]. At lower frequencies, this attenuation decreases significantly due to reduced conductive and dielectric losses. For example, at 403 MHz, the attenuation in small-intestine tissue reduces to approximately 3.34 dB/cm, while adipose tissue exhibits attenuation below 0.28 dB/cm [62].

This increased attenuation limits the achievable PTE through the body and may also contribute to tissue heating. Therefore, compliance with regulatory exposure limits, as described in Section 3, remains essential for patient safety. As a result, RF-based WPT is generally better suited for low-power implantable devices, whereas higher-power applications, such as retinal or cochlear implants, may require transmission levels that approach or exceed established safety thresholds. Nonetheless, RF is often used for data transfer, as operation at higher carrier frequencies inherently enables larger available bandwidths, while the low PTE is less critical as long as the signal reliably reaches the receiver.

### 5.3. Optical Coupling

Optical Wireless Power Transfer relies on light sources, such as lasers or LEDs, to deliver energy to an implanted device. By leveraging complementary metal oxide semiconductor (CMOS)-based photodiode arrays for energy harvesting, the implanted receiver can be made extremely compact, enabling a high degree of miniaturization [6]. Despite this advantage, optical approaches generally exhibit a much lower PTE through biological tissue compared to alternative WPT techniques (see Table 2). This limitation arises primarily from the strong absorption and scattering of light in biological tissue. Moreover, optical WPT is very sensitive to lateral and angular misalignment between the external transmitter and the implanted receiver. In addition, tissue safety remains an important topic of ongoing research for optical WPT in implantable applications [6].

Optical coupling has also been explored for wireless data transmission using intensity-modulated visible or near-infrared light. However, strong tissue absorption and scattering, combined with high sensitivity to misalignment and motion, lead to unstable channel conditions [10].

### 5.4. Acoustic Coupling

Acoustic (ultrasound-based) Wireless Power Transfer utilizes mechanical pressure waves to transmit energy through biological tissue. Because the speed of sound in biological media is much lower than that of RF waves, the wavelength is shorter at a given frequency, and similar beam directivity can be achieved at lower operating frequencies for a transducer of the same size [7]. Since acoustic attenuation in tissue generally increases with frequency, operation at lower frequencies reduces propagation losses and can improve power transfer efficiency. Alternatively, the operating frequency can be kept fixed while proportionally reducing the transducer dimensions, maintaining the same directivity. This makes it a promising candidate for powering deeper implants.

However, when acoustic energy is transmitted from an external source into the body, significant attenuation occurs at the air–tissue interface, where a large portion of the incident acoustic energy is reflected due to the strong acoustic impedance mismatch. For audible sound (20 Hz–20 kHz), this interface loss is normally compensated by the eardrum and auditory ossicles, which mechanically amplify pressure vibrations before they reach the inner ear. However, acoustic WPT operates at ultrasound frequencies (>20 kHz), for which the vibration amplitudes are extremely small. As a result, the eardrum and ossicles cannot effectively respond to or amplify these vibrations, since they are mechanically optimized only for audible frequencies. Therefore, these anatomical structures cannot effectively transfer power at ultrasonic frequencies, making acoustic WPT unsuitable for cochlear and retinal implants. However, acoustic-based WPT is effectively used in other deep implants such as gastrointestinal capsules, cardiac devices, and neural stimulators, where its ability to penetrate tissue and focus energy is advantageous [6].

In addition to power transfer, acoustic coupling has been investigated for wireless data transmission using acoustic backscatter or modulation of reflected pressure waves. While such approach can support low-power telemetry in controlled settings, literature indicates that acoustic wireless data transmission remains limited by strong sensitivity to alignment, tissue heterogeneity, motion-induced fading, and restricted achievable data rates [10].

### 5.5. Capacitive Coupling

Capacitive power transfer offers several advantages over inductive coupling, including low cost, reduced sensitivity to angular misalignment, and negligible eddy-current losses. In implantable applications, however, capacitive coupling is typically implemented using electrodes rather than well-defined parallel plates, with the intervening biological tissue acting as a lossy dielectric medium [5]. Achieving sufficient coupling therefore requires relatively large electrodes, which directly conflicts with the stringent miniaturization constraints of implantable devices [6]. Moreover, the achievable power transfer efficiency is highly sensitive to the separation distance and to the relative placement of the electrodes, decreasing rapidly as this distance increases. While operation at higher frequencies can partially mitigate this limitation by increasing the effective coupling capacitance, it simultaneously increases tissue losses and raises concerns related to safety and electromagnetic exposure [6]. Capacitive WPT systems also often employ resonant compensation networks, typically consisting of inductors that resonate with the coupling capacitances [64]. Especially in implantable applications, where the coupling capacitances between the transmitter and receiver are typically small (often in the pF range), such compensation can significantly improve the achievable power transfer and efficiency.

Capacitive coupling has also been explored for wireless data transmission through body-coupled electric fields. However, as reviewed by Lin et al. [10], capacitive wireless data transmission is highly sensitive to electrode placement, inter-electrode distance, and variations in tissue permittivity, which result in unstable channel characteristics and limited achievable data rates.

## 6. Candidate Technologies

Table 3 gives a qualitative comparison of above described wireless technologies with respect to the application-level metrics defined in Section 4. This comparison highlights that only a limited subset of technologies is capable of meeting these combined requirements.

For Wireless Power Transfer (WPT), resonant inductive coupling clearly emerges as the most suitable technology. It combines high power-transfer efficiency (also highlighted by Table 2) with favorable interaction with biological tissue and compliance with electromagnetic safety constraints, while remaining compatible with the short transmission distances typically encountered in transcutaneous links. Alternative approaches, such as capacitive, optical, and RF-based power transfer, are fundamentally limited by low efficiency, excessive tissue attenuation, or strict exposure constraints, and are therefore not considered further for high-power implant operation.

For Wireless Data Transfer (WDT), two complementary approaches are relevant. Antenna-based RF links stand out due to their inherently wide bandwidth and reduced sensitivity to angular and lateral misalignment, enabling reliable high-rate communication through biological tissue. In addition, resonant inductive coupling can also be employed for data transfer, which is particularly attractive in highly miniaturized implants, as it allows reuse of the power transfer interface and avoids the need for separate antenna structures, introducing a trade-off between achievable data rate and overall system size and complexity.

To conclude, based on this analysis, only the following technologies are considered further in this work: coil-based resonant inductive coupling for Wireless Power Transfer, and both coil-based and antenna-based solutions for Wireless Data Transfer. The highlighted regions in Table 3 visually summarize this.

The following subsections therefore delve further into the basic operating principles of both technologies. Subsequently, commonly used frequency bands and their associated design trade-offs are discussed. In later sections, the metrics introduced in Table 3 are revisited and different optimization techniques are subsequently outlined to meet the stringent requirements of cochlear and retinal implant applications.

### 6.1. Basic Principles of Coil-Based Resonant Coupling Systems

**Figure 4:**
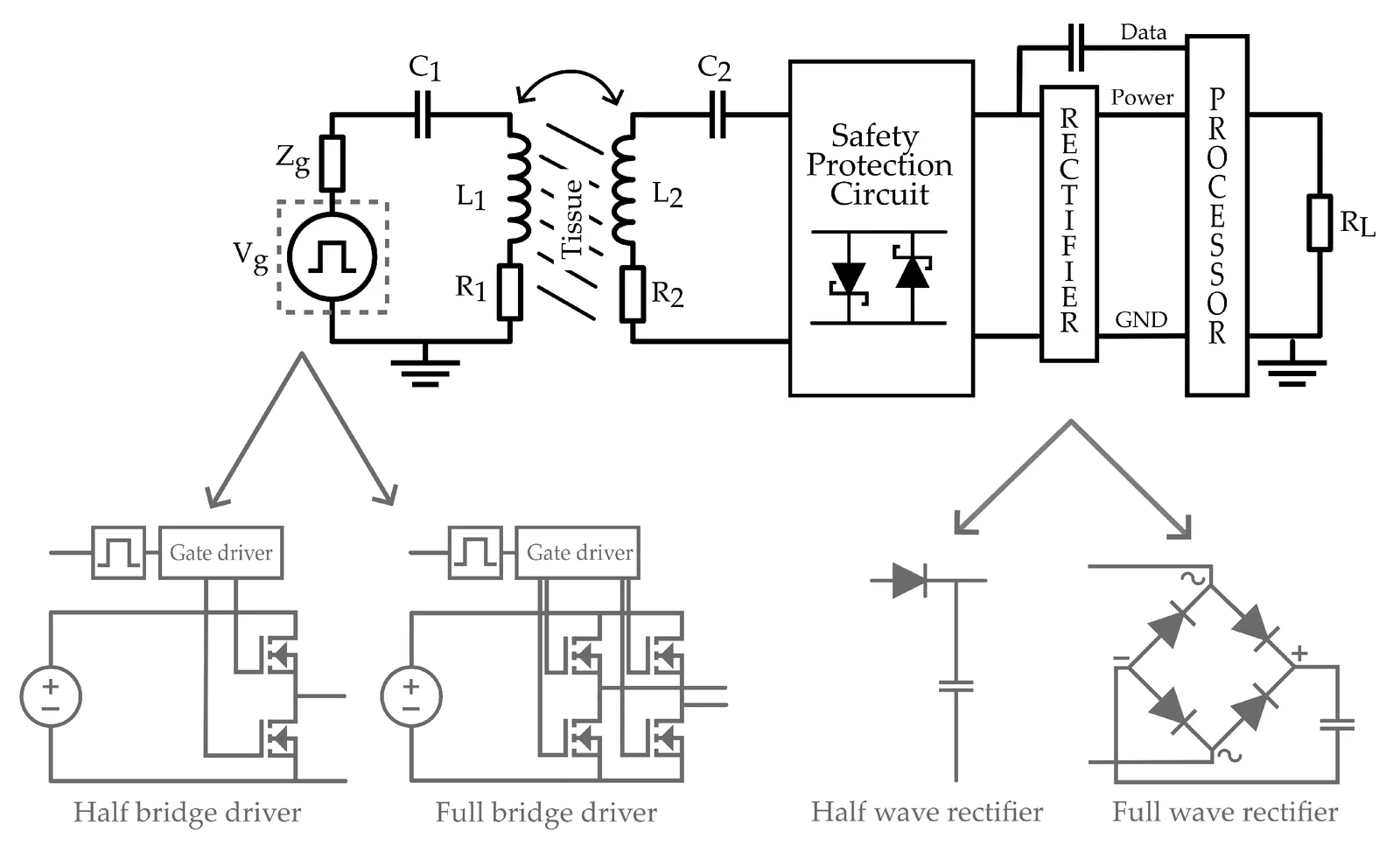
Block diagram of a resonant inductively coupled wireless power and data transfer system [69].

Figure 4 illustrates the basic architecture of a resonantly coupled wireless link for power and data transfer. The operation and performance of such a system are governed by several fundamental electromagnetic principles and design parameters, which are summarized below.

- Magnetic fields: As described by Ampere’s law, when an alternating current *I* flows through a coil, it generates a time-varying magnetic field *B*, which propagates around the coil. The magnetic field lines, associated with the magnetic flux $\Phi_{b}$, form closed loops around the current-carrying wire [67].
- Mutually coupled coils: The generated magnetic flux from coil $L_{1}$ enters the vicinity of another coil $L_{2}$, causing it to induce an electromotive force (EMF) due to the changing magnetic field. This phenomenon is governed by Faraday’s law of EM induction [67]:
  (3) $$E=-\frac{d\Phi_{B}}{dt}$$
  where E denotes the induced time-varying EMF and $\Phi_{B}$ the changing magnetic flux through the secondary coil. This EMF, in turn, drives a changing current through the second coil, which gives rise to a time-varying magnetic field *B*, given by the Maxwell–Ampère law:
  (4) $$\oint_{\partial S}\vec{B}\;\cdot \;d\vec{\ell }=\mu_{0}\int_{S}\vec{J}\;\cdot \;d\vec{A}+\mu_{0}\epsilon_{0}\frac{d}{dt}\int_{S}\vec{E}\;\cdot \;d\vec{A}$$
  where $\vec{B}$ is the magnetic flux density, $\vec{J}$ is the current density, $\vec{E}$ is the electric field, $\mu_{0}$ is the permeability of free space, and $\epsilon_{0}$ is the permittivity of free space. Furthermore, *S* denotes a surface through which the enclosed current and electric flux are evaluated, while ∂S is the closed contour bounding this surface. The quantities $d\vec{A}$ and $d\vec{\ell }$ represent infinitesimal surface and line elements of *S*, respectively.
  This time-varying magnetic field can in turn interact with $L_{1}$, thereby completing the mutual coupling mechanism.
  The strength of this coupling is described by the mutual inductance *M*, which is related to the inductance of the coils $L_{1}$ and $L_{2}$ through the coupling factor *k* [6]:
  (5) $$M=k\sqrt{L_{1}\;\cdot \;L_{2}}\;.$$
- Secondary effect: A time-varying magnetic field inherently gives rise to an induced electric field, as described by Faraday’s law of EM induction, given by Equation (3). Since inductive WPT typically operates at relatively low frequencies, the magnitude of this induced electric field remains limited. Nevertheless, it must be considered when assessing compliance with safety and exposure limits.
- Inductance: The inductance *L* of a coil depends on its geometry, number of turns, conductor dimensions, magnetic environment, … and directly influences the resonant frequency and *Q*-factor (and thus efficiency) of the system.
- Quality factor: The quality factor *Q* of a coil is defined as
  (6) $$Q=\frac{\omega L}{R}$$
  where ω is the angular frequency, *L* is the coil inductance, and *R* is the effective series resistance of the inductor. A higher *Q* in an inductive WPT system indicates reduced energy dissipation per cycle and therefore leads to improved PTE.
- Loaded quality factor: In practical systems for implants, the effective quality factor is reduced due to the load of the receiver circuitry, surrounding tissue, and additional components that introduce extra losses. When a coil is integrated into a complete circuit, its quality factor is therefore influenced by all these factors and is referred to as the loaded quality factor.
- Power Transfer Efficiency (PTE): The PTE η is defined as the ratio of the power delivered to the receiver and the total power consumed by the system:
  (7) $$\eta =\frac{P_{RX}}{P_{TX}+P_{RX}}\;.$$
  In a two-coil inductive WPT system, various compensation topologies can be employed (e.g., series–series, series–parallel, parallel–series, and parallel–parallel). In general, the PTE is primarily governed by the magnetic coupling factor *k* and the quality factors of the transmitter and receiver coils ($Q_{\mathrm{TX}}$ and $Q_{\mathrm{RX}}$). Under the assumption of resonance and optimal load matching, the maximum achievable PTE, based on the loaded quality factors, can be approximated as [6,7]:
  (8) $$\eta_{\mathrm{TX}\to \mathrm{RX}}=\frac{k^{2}Q_{\mathrm{TX}}Q_{\mathrm{RX}}}{1+k^{2}Q_{\mathrm{TX}}Q_{\mathrm{RX}}}$$
  An alternative expression for the overall optimal efficiency, which accounts for the power delivered to the load, is given by [70]:
  (9) $$\begin{array}{cc} \eta & =\eta_{\mathrm{TX}\to \mathrm{RX}}\;\cdot \;\eta_{\mathrm{RX}\to \mathrm{RL},\mathrm{ac}}\\ \; & =\frac{k^{2}Q_{\mathrm{TX}}Q_{\mathrm{RX}}}{{\left(1+\sqrt{1+k^{2}Q_{\mathrm{TX}}Q_{\mathrm{RX}}}\right)}^{2}} \end{array}$$
  In this formulation, $Q_{\mathrm{TX}}$ and $Q_{\mathrm{RX}}$ denote the unloaded quality factors, providing a more comprehensive description of the achievable system efficiency.

### 6.2. Basic Principles of Antenna-Based Systems

**Figure 5:**
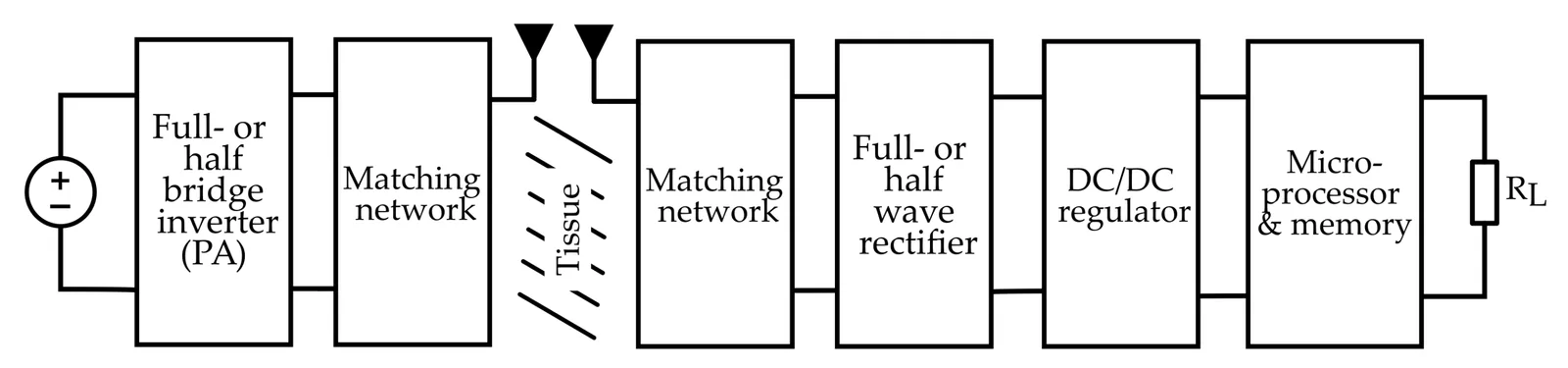
Block diagram of a basic RF coupled wireless power and data transfer circuit [69].

The basic architecture of an antenna-based wireless link is shown in Figure 5. The fundamental concepts and key performance metrics of such systems are summarized below.

- Electric fields and secondary magnetic fields: Antennas operate by driving an alternating current through a conductive structure. This causes electric charges to accumulate at the ends of the conductor, forming an oscillating electric dipole, consistent with Gauss’s law:
  (10) $$\Phi_{E}=\oint_{S}E\;\cdot \;dA=\frac{Q_{\mathrm{enclosed}}}{\varepsilon_{0}}$$
  where $\Phi_{E}$ denotes the electric flux through the closed surface *S*, and $Q_{\mathrm{enclosed}}$ is the total charge enclosed by that surface. The time-varying magnetic flux $\Phi_{B}$ generated by this oscillating dipole induces a time-varying electric field, which in turn produces time-varying magnetic fields according to Faraday’s and Maxwell–Ampère laws respectively (see Equations (3) and (4)). These coupled perpendicular time-varying electric and magnetic fields detach from the antenna and propagate through space as EM waves.
- Radiation pattern and efficiency: The radiation pattern of an antenna describes the distribution of radiated power in space. Efficient antennas are designed to focus the radiated energy in a specific direction (directivity) while minimizing losses.
- *Q*-factor, BW and gain: The quality factor of an antenna characterizes the narrowness of its resonance bandwidth. In contrast to inductive links, which benefit from a high *Q* to efficiently store and transfer energy, antennas are designed to radiate power. As a result, antennas inherently exhibit a lower *Q*-factor, corresponding to a broader bandwidth and thus supporting higher data rates. This relationship is captured by
  (11) $$Q=\frac{\omega P_{\mathrm{stored}}}{P_{\mathrm{radiated}}+P_{\mathrm{loss}}}\propto \frac{1}{\mathrm{BW}}\;.$$
  When designing an antenna, it is of interest to get an idea of the maximum achievable bandwidth and gain, as these quantities provide a benchmark against which practical designs can be evaluated.
  First, fundamental limits on antenna performance can be obtained by examining the minimum achievable *Q* (and therefore maximum BW) for a given electrical size. In this context, Chu’s limit establishes a lower bound on the *Q*-factor of an electrically small antenna enclosed by a sphere of radius *a* [71]. For the fundamental, lowest order, radiating mode (N=1), corresponding to dipole-like radiation, this bound can be expressed as:
  (12) $$Q_{\min,\;N=1}=\frac{1}{{\left(ka\right)}^{3}}+\frac{1}{ka}$$
  while for higher-order radiation modes (e.g., N=2), this yields:
  (13) $$Q_{\min,\;N=2}=\frac{2}{ka}+\frac{6}{{\left(ka\right)}^{3}}+\frac{18}{{\left(ka\right)}^{5}}$$
  where $k=\frac{2\pi }{\lambda }$ is the wavenumber. This shows that higher-order radiating modes yield larger minimum *Q* values, implying a reduced achievable bandwidth. Consequently, Chu’s limit defines a fundamental lower bound on the quality factor and, equivalently, an upper bound on the bandwidth that can be achieved by an antenna of a given electrical size (i.e., ka) and operating mode.
  In addition to bandwidth, the achievable antenna gain can be evaluated. Antenna gain quantifies how effectively radiated power is concentrated in a given direction relative to an isotropic radiator. Formally, it is defined as the ratio of the radiation intensity in a given direction to the radiation intensity that would be obtained if the same total power were radiated isotropically over all directions [72]. This can be expressed as [72]:
  (14) $$G\left(\theta ,\phi \right)=\frac{4\pi r^{2}S_{r}\left(\theta ,\phi \right)}{P_{\mathrm{rad}}}$$
  where $S_{r}\left(\theta ,\phi \right)$ is the radial component of the time-averaged Poynting vector (i.e., the far-field power density ρ(θ,ϕ)), and $P_{\mathrm{rad}}$ is the total radiated power. Using this definition, the far-field power density radiated by a transmitting antenna follows directly from spherical spreading:
  (15) $$\rho \left(\theta ,\phi \right)=\frac{P_{\mathrm{rad}}}{4\pi r^{2}}\;G\left(\theta ,\phi \right)\;.$$
  From a receiving perspective, the power received by an antenna illuminated by an incident wave, denoted as $P_{\mathrm{rec}}$, is proportional to its effective aperture:
  (16) $$P_{\mathrm{rec}}=\rho \left(\theta ,\phi \right)\;A_{e}\left(\theta ,\phi \right).$$
  where $A_{e}\left(\theta ,\phi \right)$ denotes the effective aperture.
  Combining the radiated- and receive-mode descriptions (Equations (15) and (16) respectively) therefore relates the received power to the antenna gain and effective aperture:
  (17) $$P_{\mathrm{rec}}=\frac{P_{\mathrm{rad}}}{4\pi r^{2}}G\left(\theta ,\phi \right)A_{e}\left(\theta ,\phi \right)\;.$$
  Using antenna reciprocity, this relation must remain invariant when interchanging transmit and receive operation. In free space, this invariance is formally captured by the Friis transmission equation, which relates the transmitted and received power between two antennas separated by a distance *r*:
  (18) $$P_{\mathrm{rec}}=P_{\mathrm{rad}}\;G_{t}\left(\theta ,\phi \right)\;G_{r}\left(\theta ,\phi \right){\left(\frac{\lambda }{4\pi r}\right)}^{2}\;.$$
  Assuming identical, matched antennas ($G_{t}=G_{r}=G$) and enforcing consistency between the two received-power expressions (Equations (17) and (18) respectively) in free space yields the fundamental gain–aperture relationship [72,73]:
  (19) $$G\left(\theta ,\phi \right)=\frac{4\pi }{\lambda^{2}}\;A_{e}\left(\theta ,\phi \right)\;.$$
  For a parabolic reflector antenna with physical diameter *d*, the maximum effective aperture is:
  (20) $$A_{e,\max}=\rho_{A}\frac{\pi d^{2}}{4},$$
  where $\rho_{A}$ is the aperture efficiency, typically bounded by $\rho_{A}≲0.82$ due to illumination taper, spill-over, and phase errors [72]. Substituting this expression into the gain–aperture relation yields the maximum gain:
  (21) $$G_{\max}={\left(\frac{\pi d}{\lambda }\right)}^{2}\rho_{A}\;.$$
  More general gain limits can be derived from a modal perspective by expanding the radiated fields into spherical wave functions. The maximum achievable gain associated with a radiating mode of order *N* is then given by:
  (22) $$G=N^{2}+2N\;.$$
  Since only mode orders satisfying N<ka correspond to propagating waves, this leads to the fundamental bound:
  (23) $$G_{\max}={\left(ka\right)}^{2}+2\left(ka\right)\;.$$
  This relation represents the maximum achievable gain in free space for an antenna of electrical size ka. In practical implantable scenarios, the realized gain is further reduced due to dielectric loading and losses introduced by the surrounding biological tissue.
- Impedance matching: Impedance matching ensures maximum power transfer at both the input and output of the antenna. At the input, the antenna is matched to its feed network to minimize reflections from the source, while at the output, it is matched to the connected load or receiver circuit to maximize power delivered to the load. At the input, the reflection coefficient $S_{11}$ quantifies the fraction of incident power reflected back toward the source and is therefore a primary metric for assessing the quality of the match at the antenna input. A commonly used related measure is the Voltage Standing Wave Ratio (VSWR), where values close to unity indicate good matching and minimal reflected power, while higher values correspond to increased mismatch losses and reduced radiation efficiency. These parameters are typically measured using a vector network analyzer (VNA), which characterizes the scattering parameters of the antenna [7]:
  (24) $$\mathrm{VSWR}=\frac{1+|\Gamma |}{1-|\Gamma |}\;\mathrm{and}\;\left|\Gamma \right|=10^{\frac{S_{11}\left[\mathrm{dB}\right]}{20}}$$
  with |Γ| the magnitude of the reflection coefficient, obtained by converting $S_{11}$ from decibel to linear scale.
- Link budget and margin: The link budget provides a systematic accounting of all gains and losses in a wireless link in order to estimate the received power at the receiver input [9]. The received power $P_{\mathrm{RX}}$, expressed in dBm, can be calculated as
  (25) $$P_{\mathrm{RX}}=P_{\mathrm{TX}}+G_{\mathrm{TX}}+G_{\mathrm{RX}}-L_{\mathrm{feed}}-L_{\mathrm{pol}}-PL$$
  where $P_{\mathrm{TX}}$ denotes the transmit power, $G_{\mathrm{TX}}$ and $G_{\mathrm{RX}}$ are the antenna gains at the transmitter and receiver, $L_{\mathrm{feed}}$ represents feedline losses, $L_{\mathrm{pol}}$ models polarization mismatch, and PL is the overall propagation path loss, all expressed in decibels.
  The link margin (LM) quantifies the robustness of the communication link and is defined as the difference between the received power and the minimum receiver sensitivity $P_{\mathrm{sens}}$ required for reliable communication [74]:
  (26) $$\mathrm{LM}=P_{\mathrm{RX}}-P_{\mathrm{sens}}\;.$$
  A positive link margin indicates reliable operation under nominal conditions, while additional margin provides resilience against fading, misalignment, and environmental variability.

### 6.3. Operation Frequency Bands

The selected frequencies, for both WPT and WDT, must account for the dielectric properties of human tissue, as these affect signal attenuation, heating, and penetration depth.

**Figure 6:**
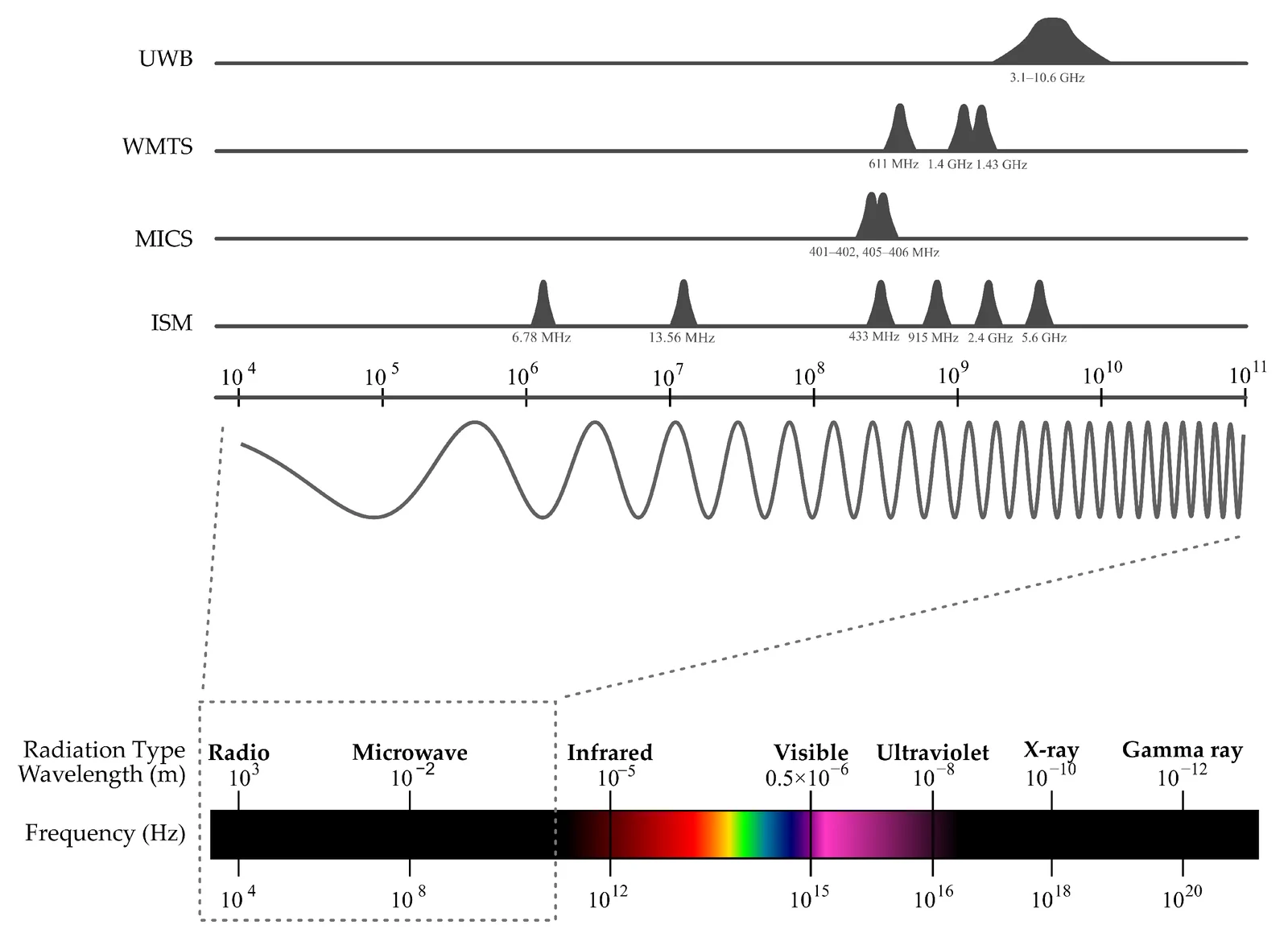
Overview of frequency bands across the electromagnetic spectrum, showing the allocation of ultrawideband (UWB),Wireless Medical Telemetry Service (WMTS), Medical Implant Communication Service (MICS), and ISM bands in relation to radiation types, wavelengths, and frequencies.

Figure 6 provides a high-level overview of the electromagnetic spectrum, highlighting frequency ranges that are most relevant for biomedical and implantable applications. In practice, the frequency bands most commonly considered for implantable wireless systems can be grouped as follows:

- ISM bands: 6.78 MHz, 13.56 MHz, 27.12 MHz, 433.92 MHz, 915 MHz, 2.45 GHz, 5.8 GHz, …
- Medical Implant Communication Service (MICS) band: 401 MHz to 402 MHz and 405 MHz to 406 MHz
- Wireless Medical Telemetry Service (WMTS) band: 608 MHz to 614 MHz, 1.395 GHz to 1.5 GHz, and 1.427 GHz to 1.4295 GHz
- ultrawideband (UWB): 3.1 GHz to 10.6 GHz

These bands will be evaluated for their compatibility with active implantable systems and for regulatory compliance [74,75].

#### 6.3.1. Coils

For coil-based systems, operating below 30 MHz is preferred as it leverages higher radio and EMC limits, allowing higher transmission power. The commonly used ISM frequencies are therefore 6.78 MHz, 13.56 MHz and 27.12 MHz. Lower frequencies provide deeper penetration and lower specific absorption rates, enhancing safety and signal robustness. In contrast, a higher frequency enables a smaller coil size and higher quality factor, which can improve link efficiency. As a result, this leads to higher PTE if the driver and rectifier can operate efficiently at the elevated frequencies. However, there is a trade-off: while a high quality factor (Q) enhances PTE, it reduces BW, making a lower *Q*-factor preferable for WDT.

#### 6.3.2. Antennas

In contrast, for antenna-based systems, the focus will be on the sub-GHz to GHz range, particularly between 403 MHz (MICS), 2.45 GHz (ISM) and 5 GHz (ISM), as these frequencies allow for physically small antenna geometries suitable for implantation. Additionally, they offer the advantage of a wide BW, which supports high data rates. A more detailed analysis of the optimal operating frequencies for the applications described in Section 2, considering antenna miniaturization, signal attenuation, and near- and far-field effects, can be found in Section 8.2.1.

## 7. Wireless Power Transfer Efficiency

Closely coupled coils, as used in conventional CI systems, provide a well-understood foundation for WPT in medical implants. However, as implants are increasingly miniaturized, achieving sufficient PTE becomes more challenging. Coil miniaturization reduces the magnetic coupling between transmitter and receiver and typically lowers the achievable inductance, leading to a reduction in PTE. To compensate for the reduced inductance, an increased number of turns is often required, which raises the series resistance and consequently degrades the quality factor *Q*. Alternatively, operating at higher frequencies can partially mitigate this. However, at higher frequencies the effective wavelength in tissue decreases, causing the coil dimensions to become a non-negligible fraction of the wavelength. As a result, part of the transmitted energy is no longer magnetically coupled, but radiated. This introduces additional losses and decreases the overall PTE. Consequently, higher transmit power is required to deliver the same amount of power to the implant, leading to increased system losses, elevated tissue heating, and greater conductive losses in both the transmitter and receiver coils. Increasing the transmit power is therefore not a viable solution. Instead, maximizing the PTE must be achieved through improvements at the coil-, circuit-, and architecture-levels, which are discussed in Section 7.1 and Section 7.2, respectively.

### 7.1. Coil Design Considerations

This subsection first focuses on the design of the transmitter and receiver coils, as their characteristics play a crucial role in determining the achievable WPT efficiency.

#### 7.1.1. Coil Design and Geometry

**Figure 7:**
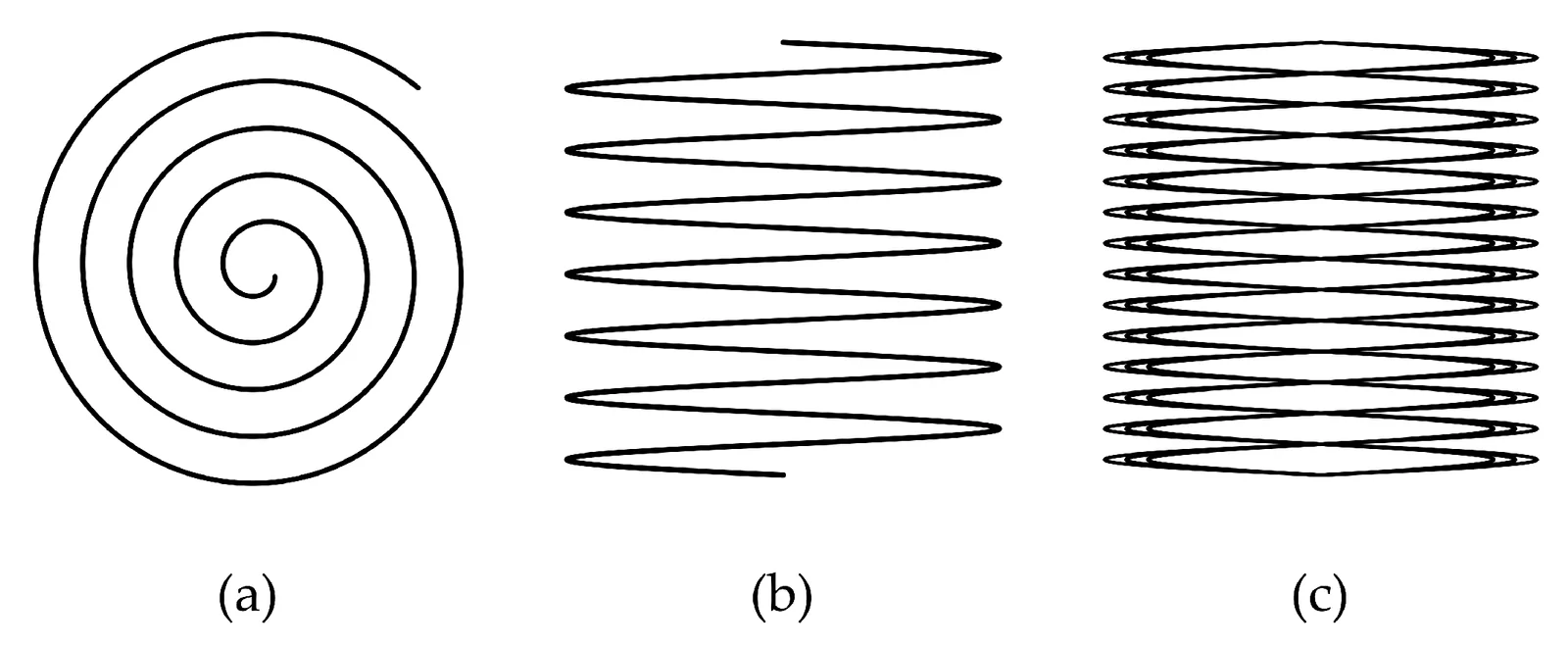
Different coil geometries: flat (**a**), solenoidal (**b**), and multi-layer (**c**).

The geometry of the coil strongly influences the magnetic field distribution, coupling coefficient, and achievable PTE. Common coil geometries include planar spiral coils, solenoidal coils, and multi-layer coil structures as shown in Figure 7.

The inductance of a large circular (single-layered) solenoidal coil is given by [76]:

(27) $$L_{\mathrm{solenoid}}=\frac{\mu_{0}\mu_{r}N^{2}A}{l}$$

where *A* is the cross-sectional area of the coil, *l* is the length of the solenoid, *N* is the number of windings, $\mu_{0}$ is the permeability of free space and $\mu_{r}$ is the relative permeability of the core material. For an air-filled coil (i.e., without a magnetic core), $\mu_{r}\approx 1$.

The inductance of a planar spiral coil, on the other hand, can be approximated using a modified version of the fundamental Wheeler’s formula [77]:

(28) $$L_{\mathrm{spiral}}=31.33\;\mu_{0}\mu_{r}N^{2}\frac{r_{\mathrm{avg}}^{2}}{8r_{\mathrm{avg}}-11w}$$

where $r_{\mathrm{avg}}=\left(r_{\mathrm{out}}+r_{\mathrm{in}}\right)/2$ is the average radius of the spiral, $w=r_{\mathrm{out}}-r_{\mathrm{in}}$ is the radial width of the coil, and *N* is the number of turns.

For all coil types, the inductance can be increased by adjusting the number of turns. In planar spiral coils, the inner and outer diameters provide additional degrees of freedom, whereas in solenoidal coils the coil diameter, axial length, and inter-turn spacing can be optimized. From Equations (27) and (28), it follows that increasing the coil dimensions or the number of turns leads to a higher inductance. However, this simultaneously increases the series resistance, as the conductor length grows. This inherent trade-off directly affects the achievable quality factor $Q\propto \frac{\omega L}{R}$ and, consequently, the overall PTE, as both depend on the relative scaling of inductance and resistance.

For solenoidal coils, the coil geometry introduces an additional design trade-off, as both the diameter and axial length influence the inductance and resistance. It has been shown that an optimal quality factor is typically achieved when the coil length is approximately equal to its diameter (l≈D) [78]. Multi-layer solenoids provide further flexibility, as the number of layers and the inter-layer spacing can be adjusted to increase the inductance within a compact footprint, albeit at the expense of increased parasitic effects.

**Figure 8:**
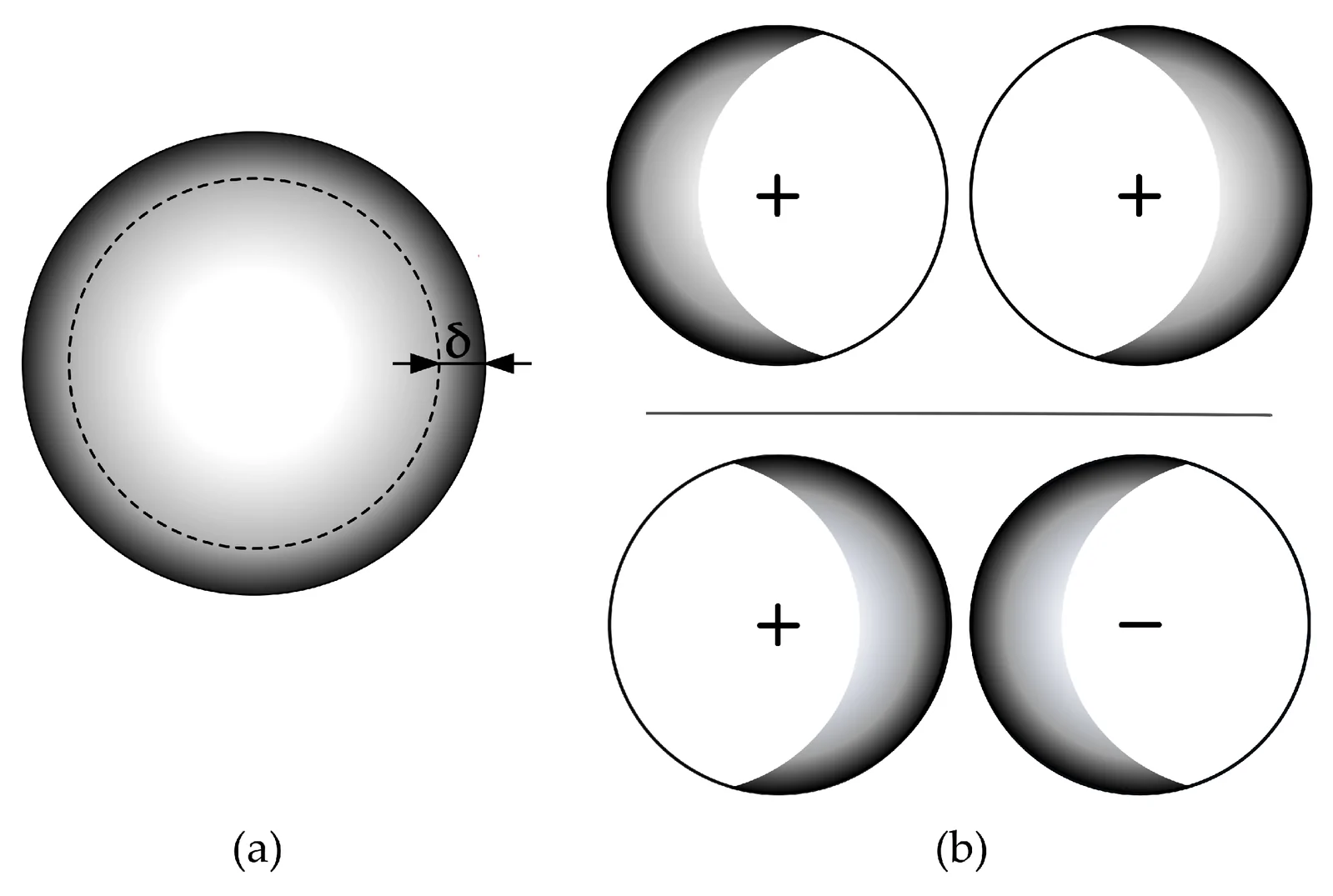
Illustration of the skin (**a**) and proximity (**b**) effects in conductive wires carrying an AC current. The + and − symbols indicate the instantaneous current direction in adjacent conductors.

Beyond geometric parameters, the conductor losses play a decisive role in the quality factor of the coil and thus the PTE. The total series resistance can be expressed as:

(29) $$R=R_{\mathrm{dc}}+R_{\mathrm{ac}}$$

where $R_{\mathrm{dc}}$ is the direct-current conductor resistance and $R_{\mathrm{ac}}$ represents the frequency-dependent resistance, primarily caused by two high-frequency loss mechanisms, namely the skin effect and the proximity effect (illustrated in Figure 8):

- Skin effect: At high frequencies, the alternating current density becomes largest at the conductor surface (skin) and decays exponentially with depth, which increases the effective AC resistance.
- Proximity effect: Proximity effect arises when time-varying magnetic fields generated by nearby conductors force the current density in a conductor to redistribute unevenly across its cross-section, typically toward one side.

Several coil design strategies can be employed to minimize $R_{\mathrm{ac}}$. Just as a thicker wire reduces the $R_{dc}$, it also reduces the $R_{ac}$ by providing a larger effective cross-sectional area for current flow. On the other hand, increasing the spacing between the turns weakens the magnetic interaction between neighboring conductors, thereby reducing the proximity effect.

For planar spiral coils, non-uniform turn spacing has even been proposed, where the inner turns are spaced farther apart than the outer turns [79]. This approach is particularly effective because the inner turns experience higher current density and contribute more strongly to magnetic coupling. By selectively increasing the spacing in this region, proximity losses can be reduced without significantly increasing size.

#### 7.1.2. Operating Frequency

The quality factor varies with frequency, but its behavior is not always intuitive. For a fixed coil geometry, the inductance *L* remains nearly constant as frequency increases. However, higher frequencies lead to stronger skin and proximity effects, which increase the effective resistance and can consequently lower the *Q*-factor.

#### 7.1.3. Ferrites

The use of ferrite materials increases the effective inductance of the coil and can steer or locally concentrate the magnetic field, thereby improving coupling and PTE. However, eddy currents and hysteresis losses in ferrite materials may introduce additional losses and heating. Moreover, due to their high magnetic susceptibility, ferrite materials can cause significant artifacts in magnetic resonance imaging (MRI). These materials locally distort the static MRI magnetic field ($B_{0}$), leading to magnetic field inhomogeneities that manifest as signal voids, geometric distortions, and shadowing effects in the reconstructed images [80]. Since many patients will require an MRI examination at some point in their lifetime, implanted systems should ideally neither degrade image quality nor be adversely affected by the MRI environment.

#### 7.1.4. Coupling Enhancement Techniques

Additional methods, such as the use of permanent magnets or leveraging the anatomical structures for improved alignment, can further increase the coupling coefficient between transmitter and receiver coils. However, the presence of such a permanent magnet in the implant during an MRI scan can cause image artifacts in the surrounding tissue, requiring appropriate precautions such as image correction techniques [81,82,83]. In addition, the strong magnetic fields in the MRI scanner can exert forces on the implanted magnet, potentially leading to displacement or rotation. In some cases, this may even require surgical removal of the magnet prior to imaging.

### 7.2. Circuit and Architecture Considerations

In addition to coil-level optimization, the overall performance of an inductive WPT system is strongly influenced by higher-level circuit and architectural design choices. While the magnetic link determines the fundamental coupling mechanism, the surrounding electronics define how efficiently energy is generated, transferred, conditioned, and ultimately delivered to the implant. These aspects are further described in the following subsections, including compensation topologies, multi-coil architectures and electronic circuits and driver implementations.

#### 7.2.1. Series/Parallel Topologies

**Figure 9:**
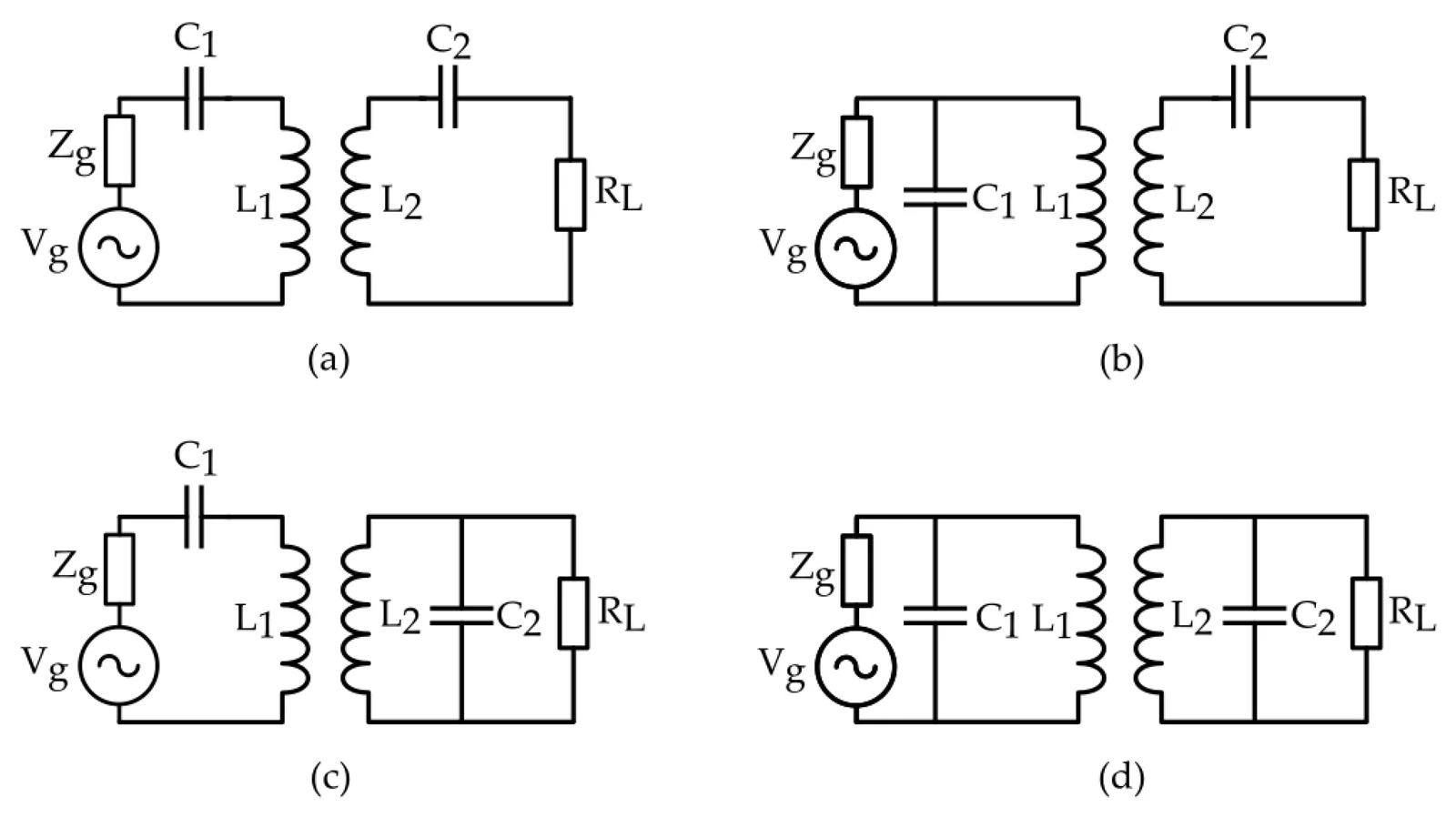
Equivalent circuit representations of the four compensation topologies: SS (**a**), PS (**b**), SP (**c**) and PP (**d**).

Compensation is achieved by adding reactive elements, typically capacitors, to the transmitter and receiver coils such that the inductive and capacitive reactances cancel at the operating frequency, as discusses in Section 5.1. The specific way in which these compensation elements are connected to the coils is referred to as the compensation topology. Several LC tank topologies are commonly used in resonant inductive WPT systems, including series–series (SS), series–parallel (SP), parallel–series (PS) and parallel–parallel (PP) configurations (all shown in Figure 9). In this notation, the first letter refers to the LC tank at the transmitter side, while the second letter denotes the LC tank at the receiver side. Here, ’S’ (series) means that the inductor (L) and capacitor (C) are connected in series, while ’P’ (parallel) means they are connected in parallel.

Each compensation topology affects impedance matching, output voltage and current characteristics, and overall system efficiency differently. The SS topology is widely used due to its simplicity and favorable operating characteristics. Series compensation at the transmitter is commonly adopted in voltage-fed WPT systems and for applications requiring larger transfer distances, whereas parallel compensation is preferred for current-source systems and when high current transfer capability is required [84]. When operated at resonance, an SS topology can provide a load-independent output current at the receiver circuit [85]. Furthermore, by operating at an appropriate off-resonant frequency, load-independent output voltage operation can also be achieved. Its main limitation is that the transferred power remains sensitive to variations in coil coupling, detuning, and load conditions. In contrast, the parallel compensation at the receiver side in the SP topology reduces this sensitivity of the transferred power to load variations and can therefore achieve high PTE under weak coupling conditions [12]. SP-type topologies are therefore frequently adopted in implantable and biomedical WPT systems. PS and PP topologies are less commonly used with conventional voltage-source transmitters because primary-side parallel compensation is more naturally associated with current-source excitation and often requires additional input inductance or current-source shaping. The PS topology may nevertheless be advantageous in applications requiring current-source characteristics or large primary currents [86]. In addition, the PP topology can maintain acceptable efficiency over a relatively wide range of load and coupling conditions and has therefore been considered for some high-power industrial applications. Its practical use, however, is often limited by low power factor, large circulating reactive currents, high input voltage requirements, and the need for larger compensation components [86].

Beyond these basic LC configurations, more advanced multi-element compensation networks, such as LCL and LCC topologies and their variants (e.g., LCL–LCL, LCC–S, LCC–LCC, …) can be employed [87]. In these networks, additional inductive and capacitive elements are connected in series and/or in parallel with the coil, forming higher-order resonant tanks. Compared to basic LC topologies, this added design freedom enables improved impedance matching, enhanced load independence, and increased robustness to coupling variations, at the cost of increased circuit complexity.

#### 7.2.2. Multi-Coil Architectures

**Figure 10:**
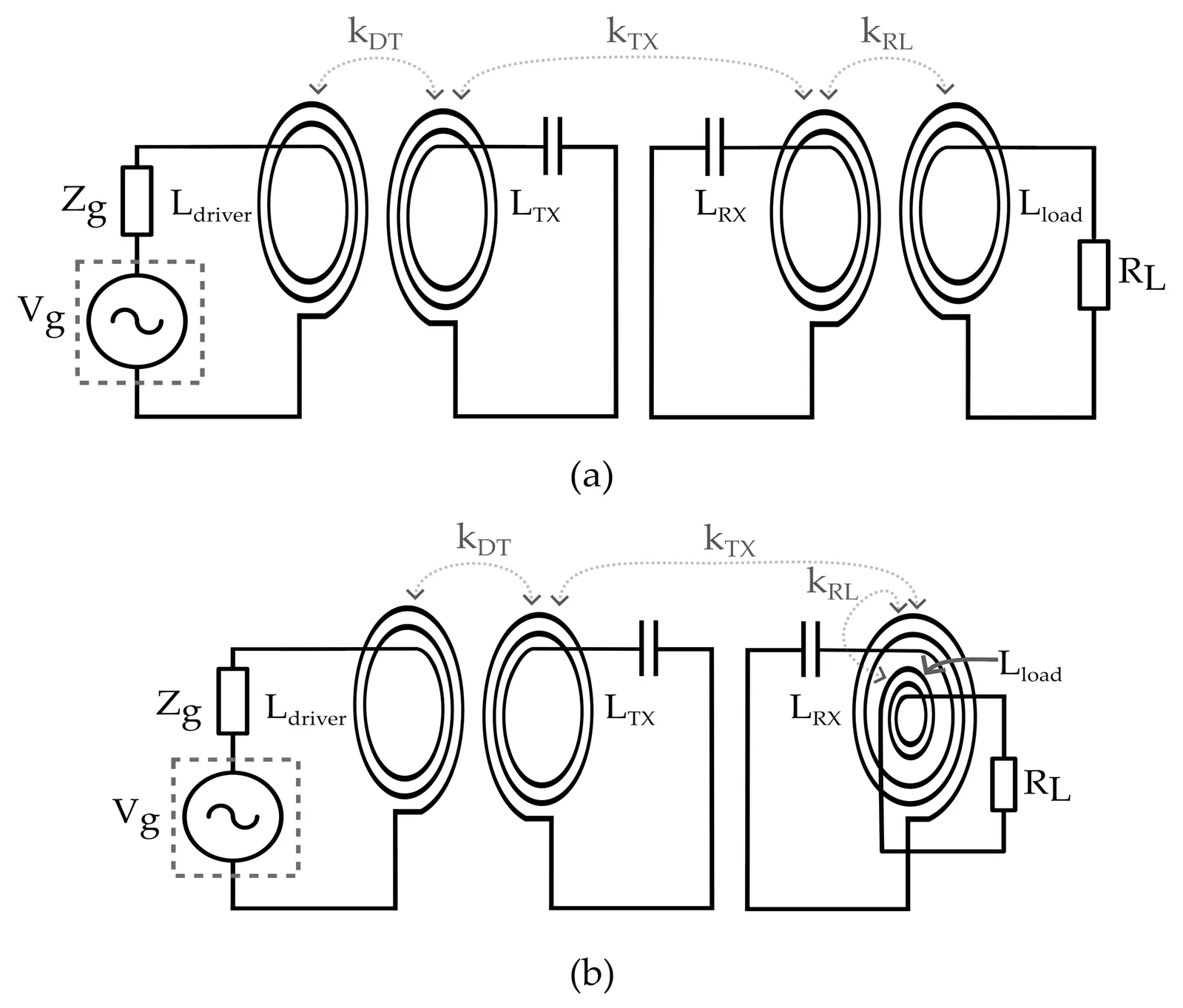
Schematic representation of the multi-coil concepts described in [88] (**a**) and [89] (**b**).

Instead of employing a single transmit and receive coil, multi-coil architectures can be used to reduce sensitivity to variations in direct TX–RX coupling. These topologies enable near-optimal PTE over a wider range of load conditions and improve tolerance to misalignment [88]. More specifically, both the transmitter and receiver circuits can be composed of two coils: a driver coil and a transmitter coil on the primary side, and a receiver coil and a load coil on the secondary side, as shown in Figure 10a. In such a configuration, the overall power transfer depends on multiple coupling coefficients, namely $k_{DT}$ between the driver and transmitter coils, $k_{TR}$ between the transmitter and receiver coils and $k_{RL}$ between the receiver and load coils. This additional degree of freedom allows the individual coupling paths to be optimized independently, thereby improving the overall coupling and PTE. While multi-coil architectures offer interesting advantages, they require additional space and increase system complexity. To mitigate the size penalty at the receiver side, Rozman et al. [89] proposed a concentric coil arrangement in which the load coil is placed inside the receiver coil. This approach (illustrated in Figure 10b) preserves the benefits of multi-coil topologies while significantly reducing the overall receiver footprint.

#### 7.2.3. Electronic Circuits and Drivers

The overall efficiency of a wireless power transfer system is strongly influenced by losses in the electronic circuits. These losses directly affect the quality factor *Q* of the resonant circuit and, consequently, the achievable PTE. Therefore, careful selection of the driver circuit and rectifier topology is required. These design choices can be broadly categorized as follows:

- At the transmitter side, the choice of the power amplifier has a major impact on the overall system efficiency. While Class-A, Class-B, and Class-C power amplifiers are relatively simple and well-understood, they suffer from low efficiencies of approximately 35%, 50%, and 65%, respectively. Consequently, these amplifier classes are generally unsuitable for power-constrained medical implant applications. For this reason, Class-D and Class-E power amplifiers are mostly employed. Class-D power amplifiers are relatively simple and robust. However, their efficiency is still typically limited to around 75% to 85% due to switching and conduction losses. In contrast, Class-E power amplifiers are specifically designed to minimize switching losses and can achieve efficiencies of up to 90% to 95%. The high efficiency of Class-E operation is achieved through zero-voltage switching (ZVS). By using an LC resonant tank tuned to a known operating frequency, the voltage across the switching device is shaped such that it ideally reaches zero at the moment the MOSFET is turned on. As a result, switching losses are minimized, which significantly improves the transmitter efficiency. In practical implementations, Class-D power amplifiers are realized as either a half-bridge or full-bridge inverter topology that drives the resonant network, as shown in Figure 4. Class-E power amplifiers are inherently single-ended and are typically implemented using a single switching device combined with a resonant network.
- At the receiver side, the rectifier circuit also contributes substantially to the total system losses. Common rectifier topologies include half-bridge and full-bridge rectifiers (illustrated by Figure 4). While half-wave rectifiers are smaller and simpler, they generally suffer from higher conduction losses and increased output ripple. In contrast, full-bridge rectifiers make use of both half-cycles of the input signal, resulting in a higher DC output voltage and improved power conversion efficiency. Alternatively, active rectifiers can be employed, where the diodes are replaced by actively controlled switches to reduce conduction losses.
  Additionally, the efficiency is largely determined by the rectifying elements. A Schottky diode with a forward voltage drop of approximately 0.3V to 0.4V, such as the SMS7630 used in [90], can be employed. This significantly reduces conduction losses compared to conventional diodes with a typical voltage drop of about 0.7 V. In low-voltage wireless power transfer systems, this choice can improve the rectification efficiency by approximately 10% to 15%, thereby increasing the overall system efficiency.

## 8. Reliable Wireless Data Transfer

Implantable devices differ in their functional requirements, which directly determine whether wireless data communication is needed. Implants whose sole purpose is to deliver therapeutic electrical stimulation, such as neural stimulators, can operate without transmitting data. By contrast, implants that restore or augment sensory perception, including retinal and cochlear implants, must convey complex visual or auditory information to the user. As a result, these systems require a reliable wireless data link to support high-fidelity information transfer.

### 8.1. Coil-Based Wireless Data Transfer

Coil-based WDT typically reuses the inductive or resonant link employed for wireless power transfer [12]. Data can be transmitted using modulation schemes such as amplitude-shift keying (ASK) and on-off keying (OOK). In ASK, logical states are represented by different amplitude levels of the carrier signal, whereas in OOK, the simplest form of ASK, the carrier is completely switched off to represent a logical low state. Due to their simple implementation and low hardware complexity, ASK and OOK are widely used in low-power implantable systems.

Other modulation schemes, such as frequency shift keying (FSK) and phase shift keying (PSK), can also be employed. Here, the information is encoded by changing the frequency or phase of the carrier signal, respectively. The simplest form of PSK, binary phase-shift keying (BPSK), uses two phase states: 0° and 180° to represent the bits ’0’ and ’1’. Quadrature phase-shift keying (QPSK) alternatively employs four distinct phase states to encode two bits per symbol (00, 01, 10, 11), thereby improving spectral efficiency. Higher-order phase- and amplitude-based schemes, such as quadrature amplitude modulation (QAM), further increase data throughput by combining phase and amplitude variations.

As both FSK and PSK maintain a constant signal amplitude, they are generally more robust against amplitude noise and power amplifier nonlinearities compared to ASK-based schemes. However, for inductively coupled links, practical constraints often favor simpler schemes, as here high-rate pulses can smear due to limited channel BW, causing intersymbol interference. Additionally, wideband signals may exceed the link’s frequency range. Consequently, schemes like OOK are often chosen as they remain reliable and easy to implement.

Finally, Load Shift Keying (LSK) can be applied through load modulation, where the load impedance of the receiver coil is intentionally varied [12]. These impedance variations modify the reflected impedance observed at the transmitter coil and can be detected as an uplink data signal.

Regardless of the chosen modulation scheme, the achievable data rate is fundamentally limited by the BW of the resonant inductive link. A high quality factor (*Q*) improves PTE but results in a narrow BW, which restricts the achievable data rate. This trade-off is illustrated by the following relation:

(30) $$Q=\frac{f_{\mathrm{res}}}{\mathrm{BW}}$$

where $f_{\mathrm{res}}$ is the resonant frequency and BW denotes the bandwidth of the link. The maximum achievable data rate is further bounded by fundamental communication limits, such as the Shannon-Hartley theorem and the Nyquist criterion, which relates the symbol rate to the available channel BW [91]. This can be expressed as:

(31) $$R_{b}\leq 2\;\mathrm{BW}\;log_{2}\left(M\right)$$

where $R_{b}$ is the data rate in bit/s, BW is the bandwidth in Hz and M the number of signal levels. In case of binary signaling (M = 2) this reduces to:

(32) $$R_{b}\leq 2\;\mathrm{BW}\;.$$

Consequently, the design goal for simultaneous wireless power and data transfer using the same coils is to select the maximum possible *Q* factor to maintain an as high as possible PTE, while still providing sufficient BW to support the required data rate.

### 8.2. Antenna-Based Wireless Data Transfer

Antenna-based WDT relies on the propagation of EM waves and is widely used for wireless communication due to its ability to support high data rates. In contrast to inductive links, which operate through near-field coupling, antenna-based systems typically exhibit lower PTE. This is generally not a limiting factor, as the received signal is amplified and demodulated by the receiver electronics, meaning that reliable communication is primarily limited by the noise floor. Furthermore, antenna-based systems can operate over longer distances and offer greater flexibility in system design. Consequently, they are well suited for WDT, as they require only sufficient received signal strength for accurate detection, rather than the efficient delivery of high power, as in WPT systems.

As shown in Section 6.2, the maximum achievable BW and gain are fundamentally constrained by the operating frequency, antenna size, and excited radiation mode. These theoretical limits provide benchmarks for practical antenna design. To approach these limits, the antenna performance must be optimized. The following subsections therefore discuss the main design parameters that determine the performance of antenna-based WDT, including operating frequency, antenna type, impedance matching, miniaturization, gain enhancement, and bandwidth enhancement.

#### 8.2.1. Operating Frequency

The optimal operating frequency represents a trade-off between several factors: miniaturization, tissue attenuation, and near- and far-field effects.

**Figure 11:**
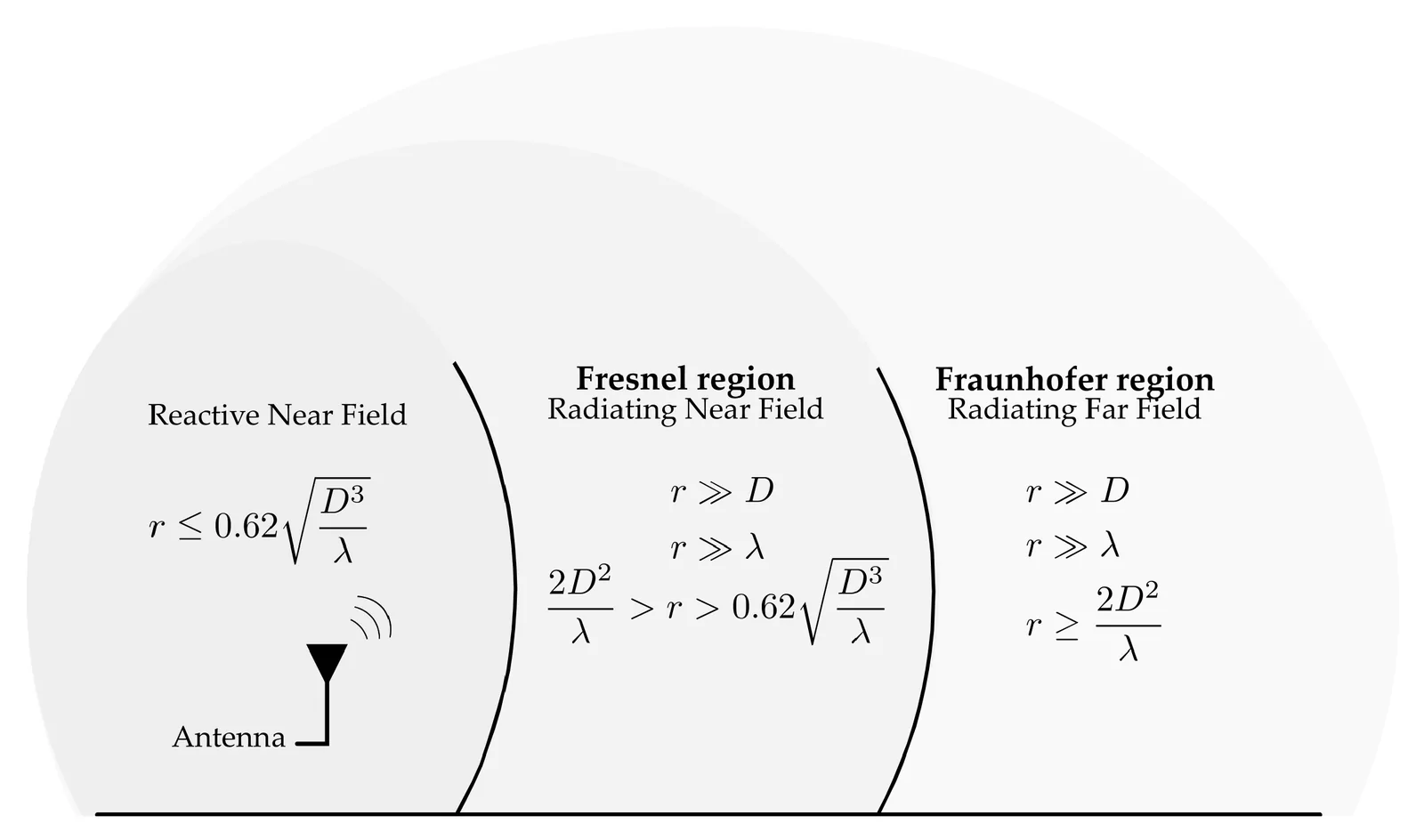
Illustration of the reactive near-field, Fresnel and Fraunhofer regions surrounding an antenna.

- Miniaturization: Higher operating frequencies allow for smaller antenna dimensions, since antenna size generally scales with the wavelength. This relationship is given by
  (33) $$\lambda =\frac{c}{f}$$
  where *c* is the speed of light in vacuum, *f* the operating frequency and λ is the wavelength. As the frequency increases, the wavelength decreases, enabling a more compact antenna design.
  However, antenna miniaturization inherently impacts performance. This trade-off is fundamentally described by Wheeler’s limit, which relates antenna bandwidth *B*, radiation efficiency η, and electrical size [92]:
  (34) $$B\;\cdot \;\eta =\frac{4\pi^{2}}{3}K_{c}\frac{V}{\lambda^{3}}$$
  where λ denotes the operating wavelength, $V/\lambda^{3}$ is therefore the normalized antenna volume, and $K_{c}$ a shape-dependent constant, typically larger than one ($K_{c}>1$). As the antenna is miniaturized, the normalized volume decreases, showing that either the BW or the radiation efficiency (or both) will be reduced. This highlights the fundamental performance penalty associated with antenna miniaturization.
- Transmission distance and field regime: The operating frequency, together with the antenna dimensions and transmission distance, determines whether the system operates in the near-field or far-field regime. This distinction is commonly described using the Fresnel and Fraunhofer regions, defined by [93]:
  (35) $$R_{1}=0.62\sqrt{\frac{D^{3}}{\lambda }}\;\mathrm{and}\;R_{2}=\frac{2D^{2}}{\lambda }.$$
  where $R_{1}$ marks the boundary between the reactive near-field and the radiating near-field (Fresnel region), and $R_{2}$ defines the beginning of the radiating far-field (Fraunhofer region). For distances $R<R_{1}$, the field is dominated by reactive electric and magnetic components, whereas for $R_{1}<R<R_{2}$ the field exhibits radiating behavior, but with a non-uniform phase front. True plane-wave propagation occurs only for $R>R_{2}$. These different field regions are schematically illustrated in Figure 11.
  In implantable systems, the transmission distance is generally much smaller than the wavelength, particularly at lower operating frequencies, resulting in near-field operation. In this regime, energy transfer is achieved through reactive coupling between the transmitter and receiver, typically relying on magnetic or electric field interactions rather than radiated waves. Increasing the operating frequency reduces the wavelength and can shift the system towards far-field operation, where communication is established through propagating electromagnetic waves.
  Both regimes exhibit distinct loss mechanisms. In the far-field regime, energy is primarily lost through propagation losses as electromagnetic waves spread and attenuate while traveling through lossy biological tissue. In the near-field regime, energy is stored in reactive fields and exchanged between the transmitter and receiver; however, part of this energy is dissipated in the surrounding medium, resulting in near-field losses. Consequently, near-field communication enables efficient short-range coupling but is highly sensitive to the relative distance, alignment, and coupling conditions between the transmitter and receiver, whereas far-field communication provides more predictable radiation behavior at the cost of increased propagation losses. The balance between these loss mechanisms depends on the operating frequency and transmission distance, and is further investigated further below.
- Tissue attenuation: The EM attenuation in biological tissue is mainly caused by two frequency-dependent loss mechanisms: electrical conductivity (σ) and dielectric loss associated with the relative permittivity ($\varepsilon_{r}$). Conductive losses arise from the motion of free ions (e.g., salts and electrolytes) driven by the electric field, where energy is dissipated as heat due to resistive losses which they experience during their movement. Dielectric losses originate from the delayed polarization response of dipolar molecules to a time-varying electric field. Due to the delayed alignment of these molecules with the time-varying electric field, energy is dissipated as heat, resulting in a reduction of the propagating field amplitude.
  Increasing the operating frequency generally leads to stronger EM attenuation in biological tissue and reduced transmission efficiency, as higher frequencies are often associated with increased electromagnetic losses and reduced penetration depth. However, the extent of this attenuation depends strongly on both the operating frequency and the specific tissue properties, and does not necessarily increase uniformly across all frequency bands. Increased attenuation is typically associated with higher tissue absorption and potential heating, which motivates the stricter regulatory limits on transmitted power at higher frequencies.

A trade-off between between the above described factors must therefore be made to determine an optimal operating frequency. For the considered implant applications, where the TX–RX separation is on the order of a few millimeters, the receiver predominantly operates in the reactive near-field region of the antenna. Increasing the operating frequency shifts the system toward the radiating near-field and eventually toward the far-field region. As a result, the dominant reactive near-field losses, which are significant at lower frequencies, are reduced. These near-field losses can be expressed for both the electric (TM) and magnetic (TE) field components as [94]:

(36) enear−fieldTM≈Reη|γ|2−iγ−1d−3Reη|γ|2−iγ−1rimplant−3

and

(37) $$e_{\mathrm{near}-\mathrm{field}}^{\mathrm{TE}}\approx \frac{{\left|\gamma \right|}^{2}+2\beta d^{-1}}{{\left|\gamma \right|}^{2}+2\beta r_{\mathrm{implant}}^{-1}}$$

where *d* denotes the implantation depth and $r_{\mathrm{implant}}$ the radius of the air sphere surrounding the antenna due to encapsulation. The complex propagation constant γ [75] is given by

(38) $$\gamma =\alpha +j\beta =j\omega \sqrt{\mu_{0}\varepsilon_{c}}$$

with complex permittivity

(39) $$\varepsilon_{c}=\varepsilon_{0}\left(\varepsilon_{r}-j\frac{\sigma }{\omega \varepsilon_{0}}\right)$$

where $\varepsilon_{r}$ is the relative permittivity and σ the conductivity of the tissue, both of which are frequency-dependent. Furthermore, ω=2πf, and $\varepsilon_{0}$ and $\mu_{0}$ denote the permittivity and permeability of free space, respectively. On the other hand, at higher frequencies, propagation losses become dominant. The electric field in a lossy medium can be expressed as [94]:

(40) $$E\left(z\right)=E_{0}e^{-\alpha z}e^{-j\beta z}$$

where α is the attenuation constant and β is the phase constant, describing the exponential decay of the field amplitude and the phase progression of the wave, respectively. Since the transmitted power is proportional to the squared magnitude of the electric field, the phase term $e^{-j\beta z}$ does not contribute to power attenuation, as $|e^{-j\beta z}|=1$. It therefore follows that

(41) $$P\left(z\right)\propto {\left|E\left(z\right)\right|}^{2}={\left|E_{0}\right|}^{2}e^{-2\alpha z}\;.$$

The term $e^{-2\alpha z}$ describes the attenuation of the wave, leading to propagation losses. For the considered geometry, this loss can be approximated as:

(42) $$e_{\mathrm{propagation}}=e^{-2\alpha \left(d-r_{\mathrm{implant}}\right)}\;.$$

The attenuation constant α is determined by

(43) α=Re{γ}

(44)     =Re{jωμ0ϵc}

(45)            =Rejωμ0ε0εr−jσωε0.

**Figure 12:**
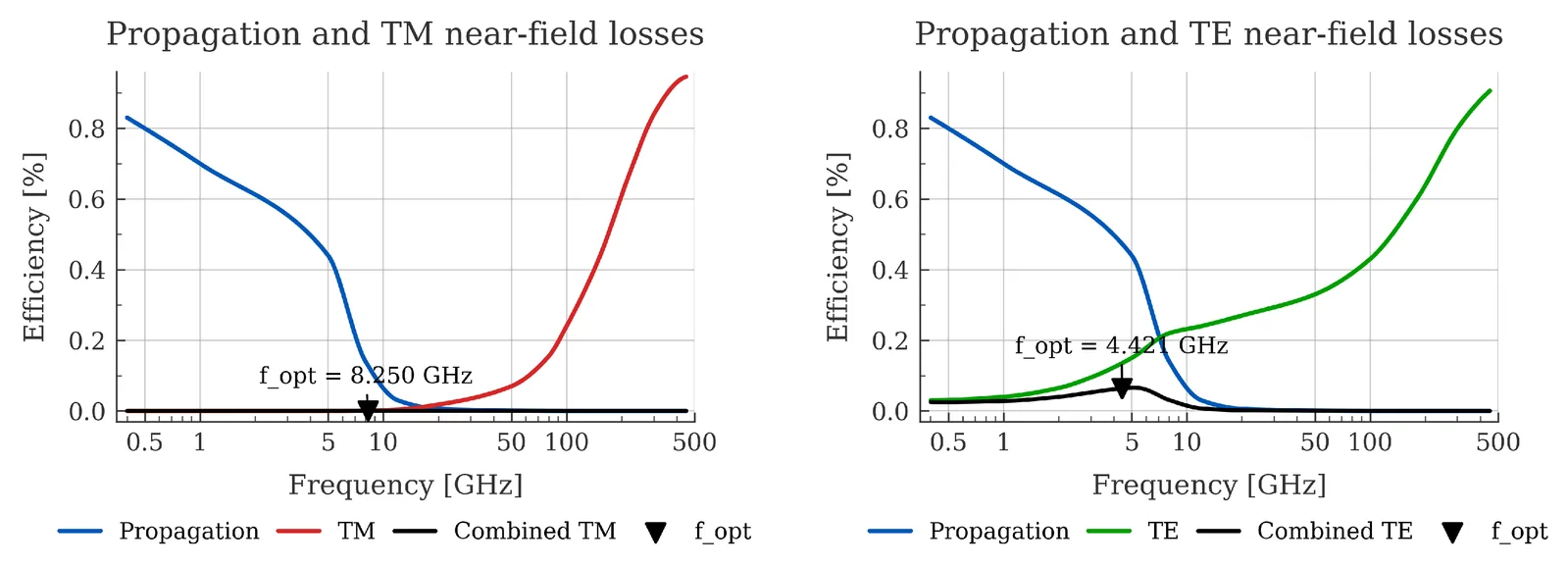
Trade-off between near-field and propagation losses as a function of frequency for a 5 mm TX-RX separation.

By combining the near-field and propagation losses, the total transmission loss can be evaluated as a function of frequency, allowing the identification of an optimal operating point. For an implant with a 5 mm TX–RX separation and an encapsulation layer that introduces an air gap of 0.1 mm around the antenna, the resulting efficiencies for both the electric (TM) and magnetic (TE) fields are shown in Figure 12.

It can be observed that increasing the frequency reduces near-field losses, while simultaneously increasing propagation losses due to stronger attenuation in tissue. The optimal operating frequency is therefore obtained by balancing these competing effects. Interestingly, the optimal frequency differs for the electric and magnetic field components: approximately 8.25 GHz and 4.42 GHz, respectively. This discrepancy originates from the fundamentally different loss mechanisms of electric and magnetic field coupling in lossy media. Electric field coupling is more strongly affected by conductive losses (through σ), whereas magnetic field coupling is less sensitive to conductivity. This is reflected in the near-field efficiency, which is lower for the electric field. Since these losses decrease with increasing frequency, electric field coupling benefits more from operating at higher frequencies, leading to a higher optimal frequency compared to magnetic field coupling. In practice, this implies that the optimal frequency depends on the dominant coupling mechanism of the antenna system. For electrically small implant antennas, where electric field coupling often dominates, a higher optimal frequency (on the order of 7 GHz to 9 GHz) is expected. However, if the system is designed to favor magnetic coupling (e.g., loop antennas), a lower optimal frequency (around 4 GHz to 5 GHz) is more appropriate.

Therefore, a practical design choice typically lies in the range of 1 GHz to 10 GHz, representing a compromise between both mechanisms and ensuring robust performance under varying conditions. This behavior is further illustrated in Table 4, where the optimal operating frequencies are listed for different TX–RX separations. It can be observed that these optimal frequencies decrease with increasing separation distance, reflecting the growing influence of propagation losses.

In addition to the near-field and propagation trade-off, a second constraint on the operating frequency arises from the attenuation of electromagnetic waves in tissue. To ensure reliable power and/or data transfer, the field strength at the receiver must remain above a minimum required level. Since electromagnetic fields decay exponentially in tissue according to $E\left(z\right)=E_{0}e^{-\alpha z}$, the attenuation coefficient α limits the maximum operating frequency and transmission distance. This behavior is characterized by the penetration depth δ=1/α, defined as the distance over which the field amplitude decreases to a factor 1/e≈0.37.

Requiring that the penetration depth exceeds the transmitter–receiver separation leads to

(46) δ≥d≈5–10mm,

which corresponds to attenuation constants on the order of

(47) $$\alpha \approx \frac{1}{\delta }=200-100\;\mathrm{Np}/m\;.$$

Applying this penetration-depth criterion using frequency-dependent tissue parameters $\varepsilon_{r}\left(f\right)$ and σ(f) yields a maximum allowable operating frequency as a function of the TX–RX separation, as included in Table 4. As expected, the optimal operating frequencies identified from the efficiency trade-off remain below this attenuation-imposed limit.

The use of gigahertz-range frequencies has also been demonstrated in the literature for similar applications. For instance, Kaim et al. [4] presented a retinal prosthesis system requiring a data rate of approximately 614 kbps. The proposed implant employs a compact circularly polarized UWB antenna with dimensions of $3\times 3\times 0.64\;{\mathrm{mm}}^{3}$, operating over multiple frequency bands: 2.45 GHz, 5.8 GHz, and 8.9 GHz. The reported TX–RX separation is 25 mm, with realized maximum gains of −20.4 dBic, −6.6 dBic, and −4.8 dBic at the respective frequency bands.

A third aspect influenced by frequency is miniaturization. Operating at frequencies in the GHz range (e.g., 5–10 GHz) results in wavelengths between 30 mm and 60 mm, which are often too large for implantable applications. However, as will be discussed in Section 8.2.4, various miniaturization techniques can be employed to mitigate this limitation.

#### 8.2.2. Antenna Type

**Figure 13:**
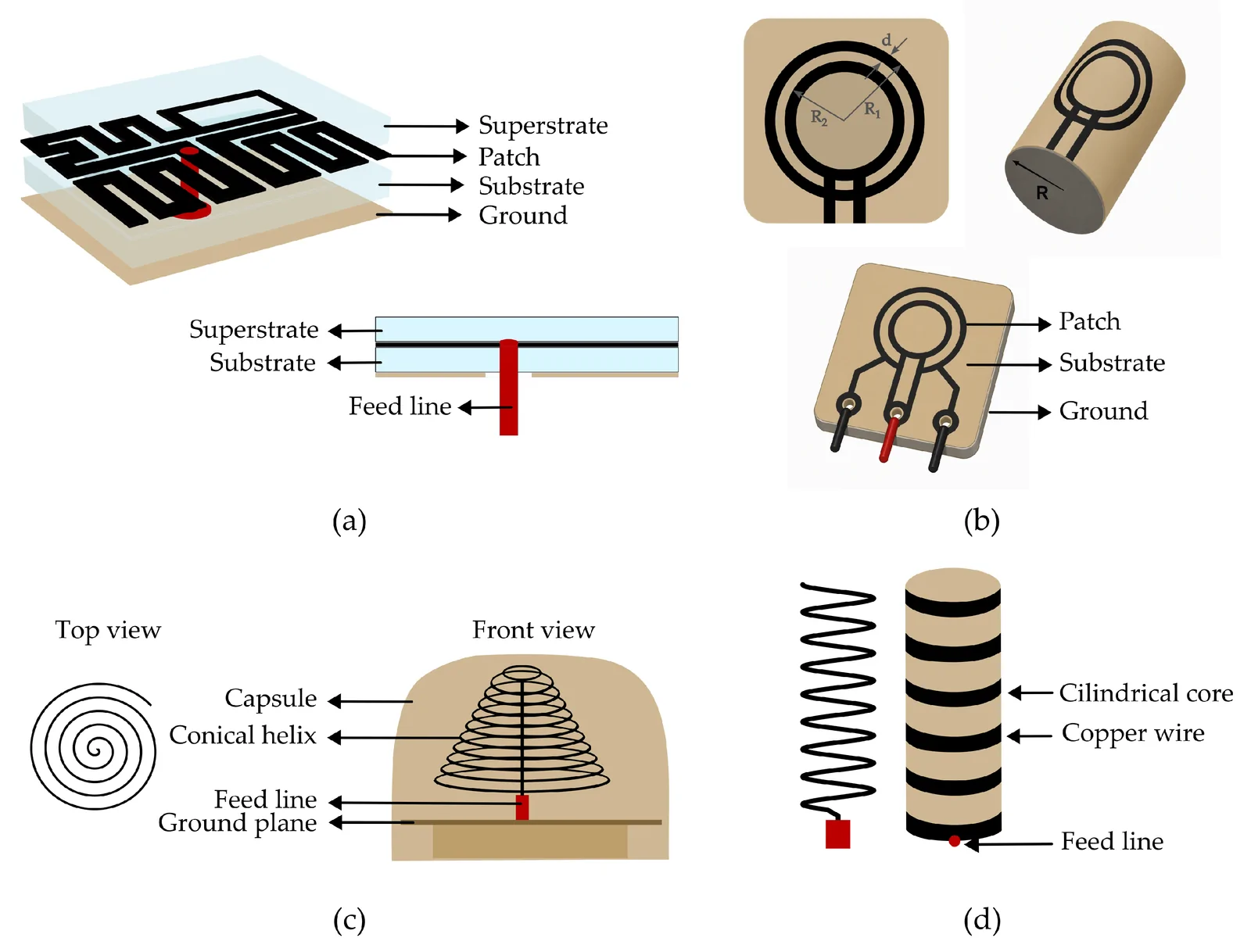
Overview of different antenna types: (**a**) a planar PCB antenna, (**b**) a flexible PCB antenna, (**c**) a spiral antenna, and (**d**) a helical antenna. Respectively, adapted from [97,98,100,101].

Implantable antennas reported in the literature can be broadly classified into planar printed circuit board (PCB) antennas and wire-based antennas [95]. Planar PCB antennas are typically realized as rigid or flexible patch structures. Rigid patch antennas are fabricated on low-loss substrates, offering stable and predictable EM performance [96,97]. Flexible patch antennas, based on polymer substrates, improve conformability with biological tissue and facilitate integration into highly miniaturized or non-planar implants, while also enabling additional miniaturization strategies (see Section 8.2.4) [98,99]. Wire-based implantable antennas on the other hand are commonly implemented in spiral or helical configurations. Spiral antennas provide compact footprints suitable for implantable devices [100], whereas helical antennas exploit three-dimensional geometries to achieve efficient radiation within limited volumes [101,102]. Other antenna types, such as whip antennas, are generally unsuitable due to their excessive size, while commercial off-the-shelf chip antennas are occasionally used for ease of integration. However, their applicability is limited by fixed operating frequencies and suboptimal performance in lossy biological tissue. An overview of these antenna types, compiled from various literature sources, is presented in Figure 13.

Recent implantable antenna designs increasingly rely on these PCB-based structures, driven by advances in miniaturization and impedance matching techniques. Depending on the application, reported designs include single-band [97,100], multi-band [103,104], and ultra-wideband antennas [98]. In addition, both linear and circular polarization schemes can be found. Circular polarization is often preferred, as it mitigates the polarization mismatch caused by random implant orientation and tissue inhomogeneity, leading to more robust wireless links [9,74,96,104]. It is typically achieved by exciting two orthogonal resonant modes with equal amplitudes and a quadrature phase difference, using techniques such as annular ground slots, shorting pins, or optimized stubs and meandered lines [4,105].

Magneto-electric (ME) antennas are a recently introduced class of MEMS-based antennas that have gained increasing interest for implantable applications. Their feasibility for wireless data transmission was first demonstrated in 2017 by Nan et al. [106]. Since then, they have been widely investigated as candidates for implantable wireless links, particularly in applications requiring extreme miniaturization [107,108].

**Figure 14:**
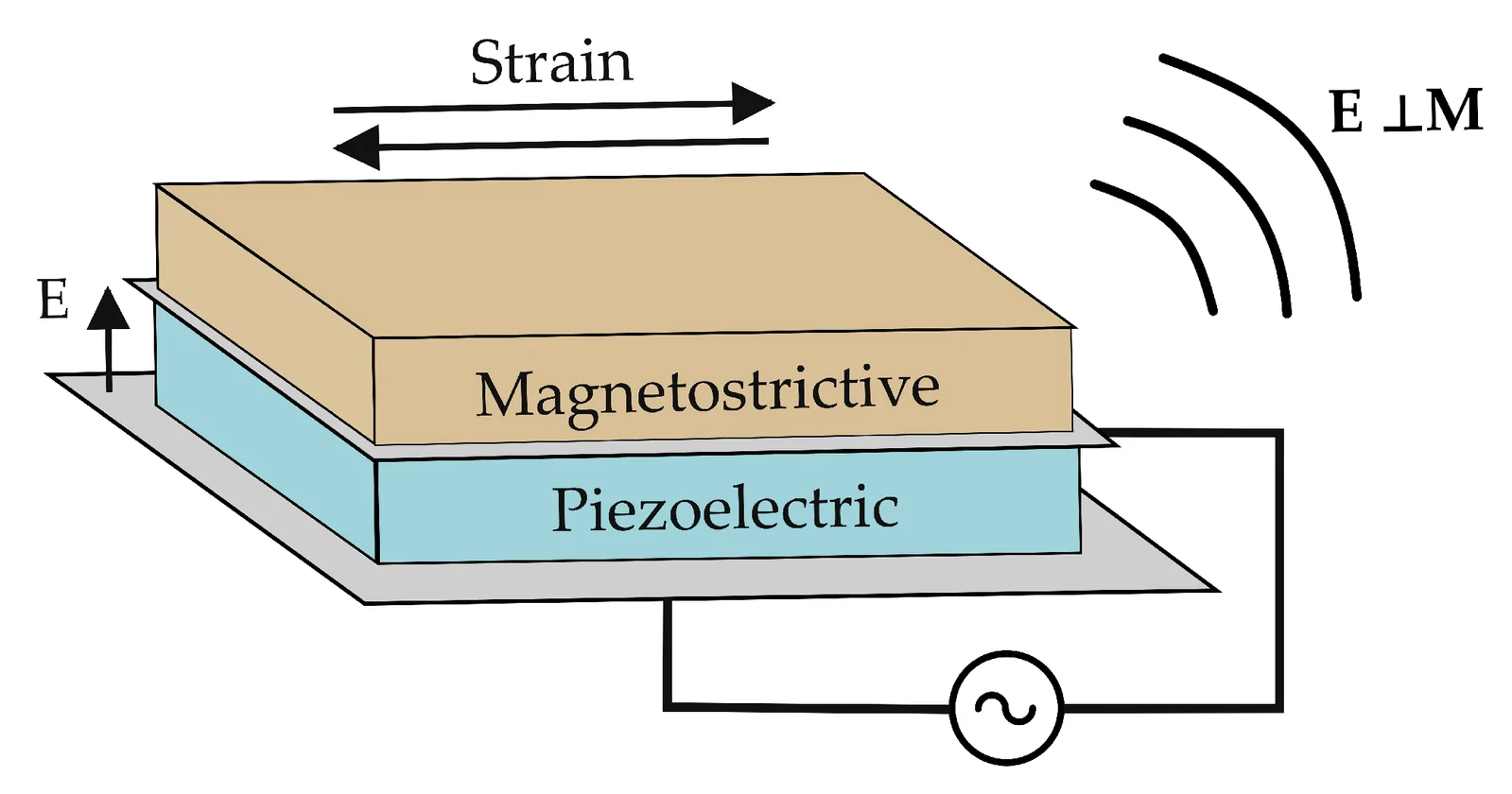
Visual representation of the working principle of a magnetoelectric (ME) antenna.

Unlike conventional antennas, ME antennas do not rely on electromagnetic wavelength-scale resonance. Instead, they exploit mechanical (acoustic) resonance in a magnetostrictive–piezoelectric structure. By applying an alternating voltage, a mechanical vibrations is induced that generates a time-varying magnetic field and, consequently, an associated electric field. This working principle is illustrated in Figure 14.

Because the antenna dimensions are set by acoustic resonance rather than the electromagnetic wavelength, ME antennas indeed enable substantial miniaturization, with device size being largely independent of the operating frequency. As a result, ME antennas can achieve volumes as small as 100 µm^3^, and antenna arrays are commonly employed to increase the achievable output power. This corresponds to a size reduction of up to four to five orders of magnitude compared to conventional electromagnetic antennas, whose dimensions are fundamentally constrained by the operating wavelength [109]. For example, Liang et al. showed that a three-element ME antenna array can generate more than three times the output voltage of a single element due to nonlinear coupling effects [110]. More recently, hybrid ME/coil receiver architectures have also been explored, combining the extreme miniaturization of ME structures with the favorable coupling characteristics of inductive links to further enhance wireless power transfer performance in biomedical implants [111]. Furthermore, ME antennas primarily radiate magnetic fields and can often be modeled as in-plane magnetic dipoles [112,113], which is advantageous in biological tissue where magnetic fields experience lower attenuation compared to electric fields.

Importantly, the performance advantages of ME antennas should be interpreted within a strongly size-constrained context. While conventional electromagnetic antennas generally provide higher radiation efficiency, gain, and bandwidth when antenna dimensions are not severely limited, their performance degrades rapidly as the antenna becomes electrically small. In such extreme miniaturization regimes, ME antennas can achieve higher effective link performance by decoupling antenna dimensions from the electromagnetic wavelength, as emphasized in recent review work [109].

Experimental studies validate this distinction across a broad frequency range. At gigahertz frequencies, Das et al. demonstrated that an ME antenna with dimensions of 250 μm× 174 μm, operating at 2.515 GHz, can achieve a PTE of 0.28% over a 3 mm distance while maintaining a 22.7 MHz bandwidth due to a high quality factor [108]. These results illustrate that ME antennas can support practical high-frequency wireless links despite operating at deeply sub-wavelength scales.

At lower operating frequencies, where magnetic near-field coupling dominates, higher effective power transfer efficiencies have been reported for strongly miniaturized systems. In the comparative analysis of [113], a 200 μm-scale ME antenna achieved an effective PTE of up to 30% at kilohertz frequencies under favorable coupling conditions. Under the same assumptions, including a magnetic coupling coefficient of 0.1% and a transmitting-coil quality factor of 4, equivalently sized spiral and solenoidal coils reached only 0.17% and 0.12%, respectively [113]. As summarized in [109], these gains reflect a fundamental advantage of these ME antennas in extreme miniaturization scenarios, where electromagnetic antennas and coils suffer severe efficiency degradation due to electrically small operation.

#### 8.2.3. Impedance Matching

As mentioned in Section 6.2, proper impedance matching is essential for maximizing antenna efficiency and gain, as impedance mismatch leads to power reflection and reduced radiation efficiency. This is particularly critical for implantable antennas, where available transmit power is limited by tissue losses and safety constraints. Impedance matching networks (which are composed of reactive elements like inductors and capacitors) can be designed by tools such as the Smith chart to compensate for the reactive part of the antenna impedance. These networks can be implemented either as discrete circuits placed adjacent to the antenna or integrated directly into the antenna structure, which is called capacitive or inductive loading techniques [114].

When implemented as external circuits, matching networks require additional physical space, but offer reduced complexity and increased design flexibility. Common topologies such as L-, T-, and π-networks provide impedance matching and allow control over the quality factor ($Q_{L}$) and, consequently, the system BW [115]. Moreover, T- and π-networks also act as higher-order filters, improving out-of-band noise suppression and matching a wider range of load impedances, thereby enabling optimal matching under varying load conditions.

Alternatively, additional inductive or capacitive elements can be integrated directly into the antenna geometry to offset the imaginary part of the impedance. In this approach, inductive or capacitive loading is used to modify the effective electrical length of the antenna, thereby compensating for its reactive impedance while simultaneously contributing to size reduction. Inductive loading has been widely applied for antenna miniaturization [116], while capacitive loading has been shown to reduce antenna size by up to 72% compared to conventional implantable patch antennas [114]. A geometry-based realization of this concept is split-ring resonator (SRR) loading, which provides an effective approach for both antenna miniaturization and impedance matching by increasing the antenna’s effective electrical length [117]. This latter one is further elaborated in Section 8.2.4.

#### 8.2.4. Antenna Miniaturization Techniques

**Figure 15:**
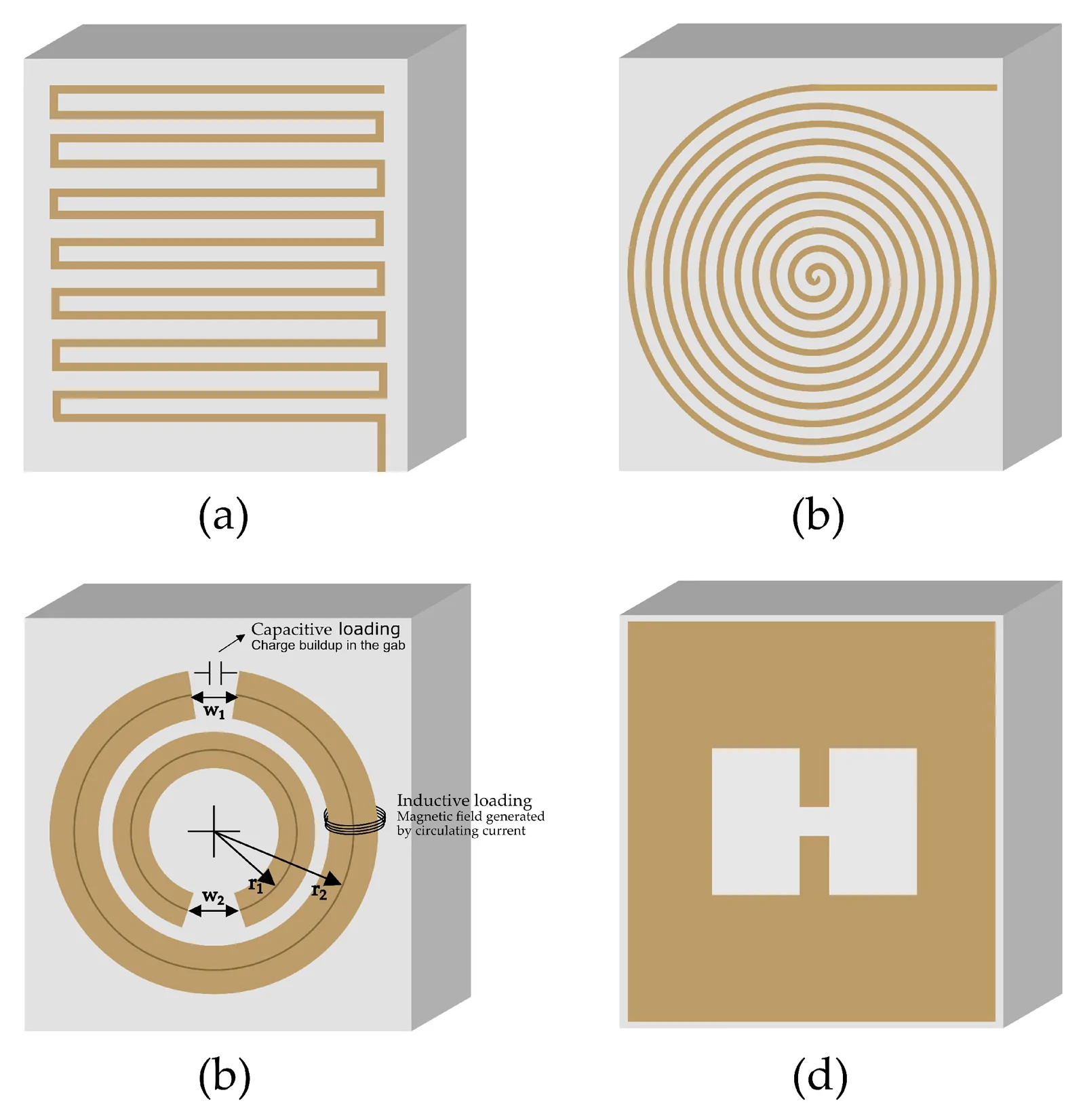
Different antenna miniaturization techniques including current-path modifications of the radiating element, such as meandered (**a**) and spiral (**b**) geometries, an SRR (**c**) that introduces additional inductive and capacitive loading, and a dumbbell-shaped DGS (**d**) that modifies the ground plane.

To reduce antenna size while maintaining acceptable performance, several design strategies can be employed [9,53]. These techniques are schematically illustrated in Figure 15.

- High-permittivity dielectric substrates ($\varepsilon_{r}$) [117]: Although high-permittivity materials introduce additional dielectric losses, they are widely exploited for antenna miniaturization. Due to the delayed polarization response of such substrates, an additional electric field component is induced that partially counteracts the applied field, effectively slowing down EM wave propagation. Since the operating frequency remains constant, a reduced propagation velocity results in a shorter wavelength, given by $\lambda_{\mathrm{eff}}=\lambda /\sqrt{\varepsilon_{\mathrm{eff}}}$. As antenna dimensions scale with effective wavelength, increasing the substrate permittivity directly enables antenna size reduction.
  Common used materials are listed by Table 5. In addition to the relative permittivity, the dielectric loss tangent (tanδ) is given. This is a critical parameter as higher dielectric losses lead to reduced radiation efficiency and increased power dissipation within the substrate. While often used materials like Rogers RO6010 can yield an improvement of 14%, substrates with an even higher permittivity like MgTa_1.5_Nb_0.5_O_6_ can reduce the size by 29% [95].

**Table 5:** Relative permittivity and loss tangent of substrate materials commonly used for antenna miniaturization.

Material	$\varepsilon_{r}$	tanδ	References
FR4	4.7	0.025	[118]
Rogers RO6010	10.2	0.0023	[97,98,103,105,119,120,121]
Rogers RO3010	10.23	0.0035	[54,96,114,116,122,123,124,125,126]
Rogers R3210	10.2	0.003	[127,128,129,130,131]
Al_2_O_3_	9.8	0.002	[132,133]
MgTa_1.5_Nb_0.5_O_6_	28	–	[134,135]

- Lengthen the effective current path [117]:
  − Current-path geometry: By increasing the electrical path length of the surface current without enlarging the physical antenna footprint, the antenna can resonate at lower frequencies, thereby enabling size reduction. Common implementations include meandered lines [95,118], spiral geometries [103], and fractal structures [95,136]. Fractal-based designs, for example, have demonstrated antenna miniaturization of up to 55.1% relative to a conventional patch antenna with the same outer dimensions [136].
  − Shorting pins: Shorting pins introduce a direct electrical connection between the radiating element and the ground plane, fundamentally altering the antenna’s boundary conditions. The shorting pin enforces a current maximum and a voltage minimum at its location, shifting the resonance from the conventional half-wavelength (λ/2) mode to a quarter-wavelength (λ/4) mode. As a result, the antenna can resonate at approximately half the frequency (i.e., double the effective wavelength) for the same physical size, allowing miniaturization.
    Planar Inverted-F Antennas (PIFAs) represent a standardized planar implementation of this shorting-pin miniaturization principle and are widely used for compact antenna designs [129,137,138]. A PIFA consists of a planar radiating element positioned above a ground plane, with a defined feed point and shorting pin. Compared to generic shorted antennas, the PIFA topology offers predictable impedance characteristics and compact planar integration. Additionally, it provides improved robustness against detuning caused by surrounding biological tissue, making it particularly attractive for implantable applications.
  − Radiator stacking: Another miniaturization approach involves stacking multiple radiating elements either vertically or horizontally, effectively trading lateral dimensions for increased thickness. In [139], two circular meandered radiators are vertically stacked, resulting in a compact antenna volume of 32.7 mm^3^. Vertical stacking techniques are commonly reported to achieve size reductions on the order of 30% to 35% [95].
- Slot-loading techniques Adding open slots in either the ground or radiating plane can enhance miniaturization, as these can increase the current path. More specifically, L-shaped or C-shaped slots on the ground plane or split-ring resonator (SRR) are commonly used [74]. This latter one is a compact structure that introduces additional inductive and capacitive loading when coupled to an antenna [53,140]. This reactive loading increases the effective electrical length of the radiator, enabling resonance at lower frequencies without increasing the physical antenna size.
- Defected ground structures (DGS): [136] Defected ground structures modify the current distribution in the ground plane by introducing intentional slots or patterns, thereby increasing the effective inductance and electrical length of the antenna. Common examples include dumbbell-shaped defected ground structures (DGS), which can be modeled as additional lumped inductive and capacitive elements. These added reactive components shift the resonance to lower frequencies, enabling antenna miniaturization. In [136] when considering the biggest dimensions of the patch antenna, using a DGS with dumbbell shape results in a miniaturization of 26.8%.
- Folding the antenna geometry: Folding the antenna increases the effective electrical length while maintaining a compact physical footprint. It has even been shown that folding the antenna slightly shifts the resonant frequency to lower values (e.g., on the order of 1%) [141]. However, the altered current distribution comes at the cost of reduced efficiency.

#### 8.2.5. Communication Framework

Antenna-based systems require a robust communication framework to ensure reliable data transfer through biological tissue. This includes a suitable frame structure (e.g., header and preamble) to enable synchronization and reliable demodulation.

Furthermore, the choice of modulation scheme (see Section 8.1) has a strong impact on robustness and power efficiency. For antenna-based implantable communication systems, FSK is most commonly employed in the literature, as it offers good bit error rate (BER) performance and is inherently robust against amplitude variations caused by misalignment and tissue absorption. ASK is generally avoided because of its sensitivity to noise and amplitude fluctuations. PSK can achieve better BER performance, but requires more complex receiver architectures and higher power consumption, making it undesirably complex.

Finally, error detection and correction mechanisms can be implemented to improve link reliability. Simple error detection schemes are often preferred, while more advanced forward error correction (FEC) techniques may be used at the cost of increased latency, complexity, and power consumption.

#### 8.2.6. Gain Enhancement

Additional efficiency improvements can be achieved through the use of high-permittivity superstrates, optimized capsule filling materials, and compatibly encapsulation layers, which help reduce impedance mismatch, detuning, and radiation losses [9].

- Superstrate: Similar to the substrate, a superstrate with high relative permittivity can shorten the effective wavelength of the EM field, enabling antenna miniaturization. In addition, the superstrate provides electrical insulation between the antenna and surrounding tissue and can help confine and direct the radiated fields, potentially leading to improved antenna gain and radiation efficiency [105].
- Capsule size: Increasing the capsule size, and thus enlarging the surrounding air gap around the antenna, reduces the interaction of the radiated fields with the lossy tissue environment. Since electromagnetic waves experience significantly lower attenuation in air compared to biological tissue, this effectively reduces both near-field and propagation losses.
  For an implant located at a depth of 5 mm and surrounded by an encapsulation layer with a thickness of 1 mm, the optimal operating frequencies shift towards lower values compared to the previously discussed case with a thinner encapsulation layer. Based on the trade-off between near-field coupling and tissue propagation losses (see Section 8.2.1), the optimal frequencies become $f_{\mathrm{opt},\mathrm{TM}+\mathrm{prop}}=4.85\;\mathrm{GHz}$ and $f_{\mathrm{opt},\mathrm{TE}+\mathrm{prop}}=2.85\;\mathrm{GHz}$. This demonstrates that, for shallow implant depths, the balance between reactive coupling losses and tissue-induced propagation losses is achieved at lower frequencies. Operating at these frequencies reduces electromagnetic attenuation in the surrounding tissue, thereby improving the overall wireless transmission efficiency and maintaining a higher received power level.
- Capsule filling: The capsule can be filled with a dielectric material, which then becomes part of the antenna’s electromagnetic environment and can reduce the dielectric mismatch between the encapsulation layer and the surrounding tissue. This provides an alternative to increasing the capsule size and introducing an air gap. High-dielectric materials such as water ($\epsilon_{r}\approx 78.4$) or glycerol ($\epsilon_{r}\approx 12$) can therefore reduce the dielectric mismatch between the encapsulation layer and the surrounding tissue [141,142]. This smoother transition in dielectric properties reduces reflections at material interfaces, allowing a larger fraction of the electromagnetic energy to couple into the surrounding tissue and improving the overall radiation efficiency of the implant.
- Encapsulation: An encapsulation material also acts like a superstrate, and thus one with a high permittivity is preferred. However, it does need to be biocompatible, and only a few of such materials are reported in literature for implants, e.g., ${\mathrm{MgTa}}_{1.5}{\mathrm{Nb}}_{0.5}O_{6}$ ($\epsilon_{r}$≈ 28) and ${\mathrm{Al}}_{2}O_{3}$ ($\epsilon_{r}$≈ 10) [141]. They are able to isolate the antenna and electronics safely from biological tissue. From an EM perspective, a suitable encapsulation layer provides a gradual transition in dielectric properties between the antenna and tissue, reducing abrupt field distortions and associated losses, possibly improving efficiency.

#### 8.2.7. Bandwidth Enhancement

Substrate materials with higher relative permittivity ($\epsilon_{r}$) are commonly used for antenna miniaturization, since they reduce the effective wavelength. However, this typically comes at the cost of reduced bandwidth. The stronger field confinement increases the antenna quality factor (*Q*-factor), resulting in a narrower resonance and therefore a smaller bandwidth (BW∝1/Q). This trade-off can be approximated as

(48) $$\mathrm{BW}\propto \frac{1}{\sqrt{\epsilon_{r}}}\;.$$

Several antenna-level techniques can be used to enhance the BW. Loop-shaped radiators and meandered DGS structures increase the effective current path length and can introduce additional resonant modes [9]. Additionally, the use of parasitic patches placed in close proximity to the driven radiator enables EM coupling that excites multiple closely spaced resonant modes, thereby significantly broadening the operational BW without increasing the antenna footprint or feeding complexity [75].

Furthermore, the literature explores multichannel communication strategies, including parallel data transmission across multiple frequency bands using dual-band, tri-band, or quad-band antennas. Such designs are typically realized through the appropriate selection of feed and via locations, with different frequency bands often allocated to uplink and downlink communication, as demonstrated in [90]. In addition, multiple-input multiple-output (MIMO) antenna systems enable the simultaneous transmission and reception of multiple data streams [9]. Besides increasing the effective data throughput, these approaches improve robustness against interference, frequency-selective attenuation, and band-specific degradation, thereby enhancing overall link reliability.

### 8.3. Coil-Versus-Antenna Selection Framework

**Figure 16:**
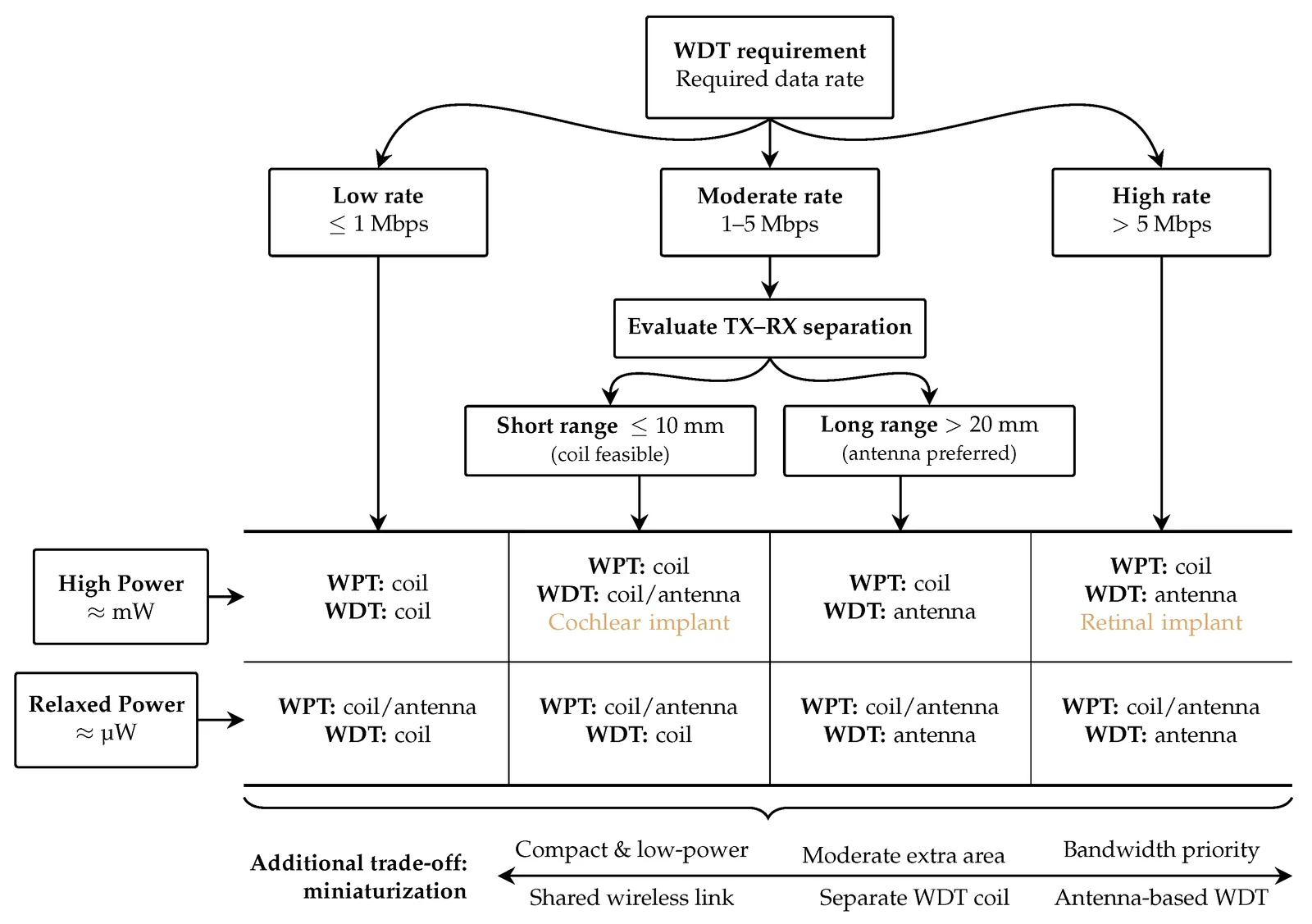
Decision framework for selecting WPT and WDT technology.

The selection of suitable WPT and WDT technologies depends on several application-specific parameters, including the required data rate, transmission distance, and implant power budget. Figure 16 therefore provides a high-level technology selection guide for implantable wireless links, based on the trends identified throughout this review.

## 9. Integrated WPT and WDT

WPT and WDT can be integrated using either a single wireless link or multiple wireless links. In a single-link architecture, power and data are transmitted simultaneously through the same physical channel, whereas in a multi-link architecture, separate channels are used for power and data transmission. Both approaches involve different trade-offs regarding system complexity, transmission efficiency, and interference management.

The simultaneous transmission of wireless power and data over a single channel is commonly studied in the wireless communications literature under the framework of simultaneous wireless information and power transfer (SWIPT) [143]. SWIPT mainly focuses on RF-based far-field systems, where a radiated waveform is used both for data communication and energy harvesting. However, as discussed previously, the power that can be extracted from RF signals is typically small, making SWIPT unsuitable for the high-power implant. Therefore, the following section provides an overview of integrated WPT and WDT architectures, focusing on the technologies introduced in the previous sections.

### 9.1. Single Wireless Power and Data Link

In a single wireless link, the same inductive channel is used for both power and data transfer. This minimizes hardware overhead and implant size, but requires careful coordination between power delivery and data communication. In this case, the data is superimposed on the power carrier by a specific modulation scheme like ASK, OOK, FSK, PSK or LSK, as explained in Section 8.1 [13]. A key drawback of this approach is that the same coils, and therefore the same quality factor *Q*, must be used for both power and data transfer. This introduces a trade-off between PTE and communication BW: a high *Q* is favorable for efficient power transfer but limits BW, whereas a lower *Q* increases BW at the expense of PTE.

To mitigate this trade-off, time-division multiple access (TDMA) can be employed. In this scheme, power and data are transmitted in separate intervals, where for power transfer a coil with high *Q*-factor is used and for data transfer the *Q*-factor is temporarily reduced (de-*Q*ing). This is achieved by adding an extra switchable resistor in parallel with the LC circuit, therefore lowering the *Q*-factor.

Another, less common approach reuses the inductive coil itself as a radiating antenna for data transmission [144]. In this case, dedicated filtering and matching networks are integrated to separate the power and data paths. Typically, a low-pass filter (LPF) extracts the low-frequency inductive power signal, while a high-pass filter (HPF) isolates the higher-frequency data signal. Alternatively, two band-pass filters (BPFs) can be employed, at the cost of increased circuit complexity. These filtering stages are combined with impedance matching networks to enable efficient transmission and reception of the data signal.

**Figure 17:**
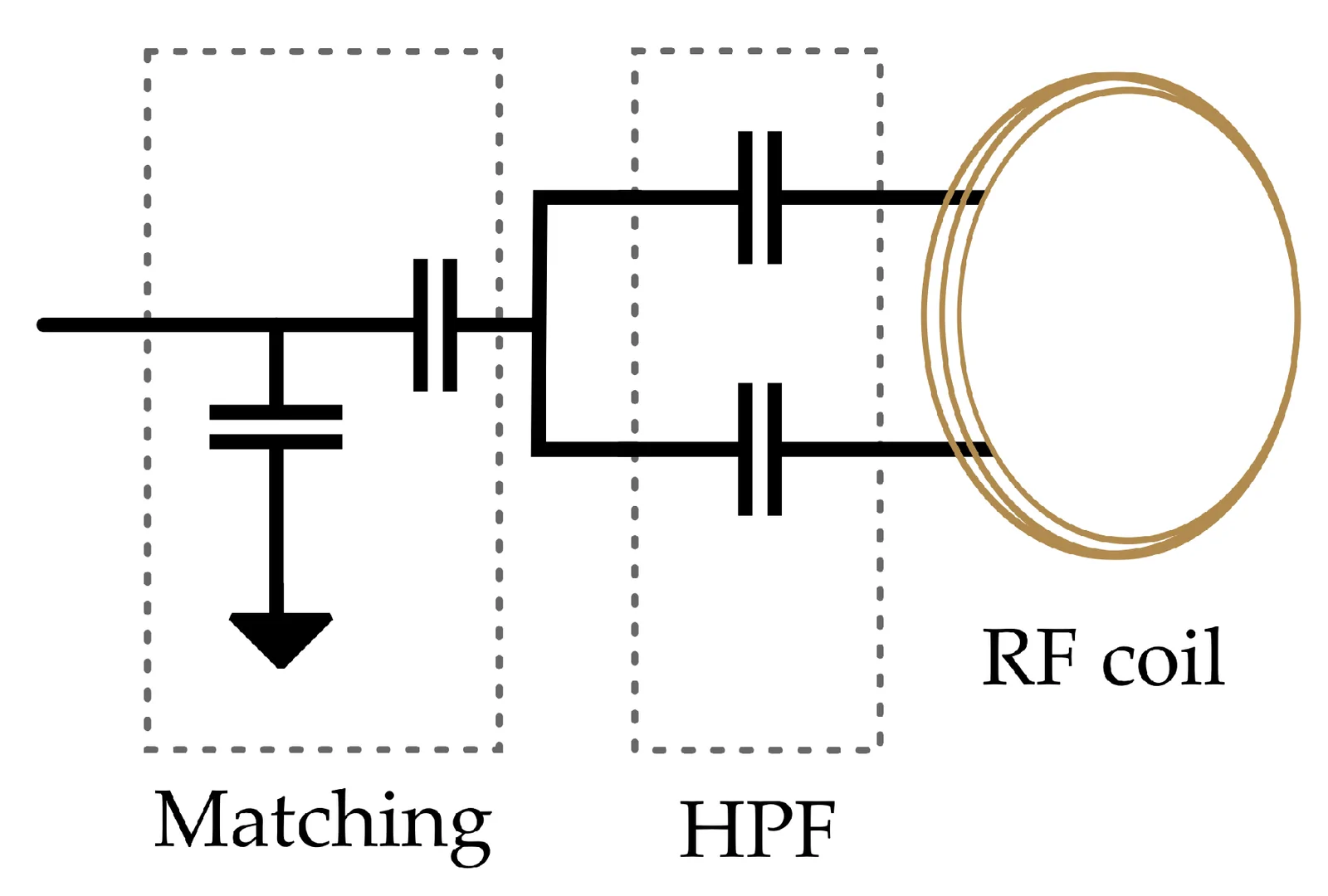
Schematic illustration of reusing the coil as an antenna by employing a HPF and an impedance-matching network to extract the data captured by the coil.

This principle is also illustrated by Figure 17. While this approach enables the reuse of a single physical structure, the additional filtering and matching circuitry may reduce the practical benefits of hardware sharing in terms of overall complexity and miniaturization.

### 9.2. Multiple Wireless Power and Data Links

In a separate-channel approach, WPT and WDT are handled by distinct physical links. Several techniques can be employed, each with their own trade-offs and design strategies to minimize mutual interference.

**Figure 18:**
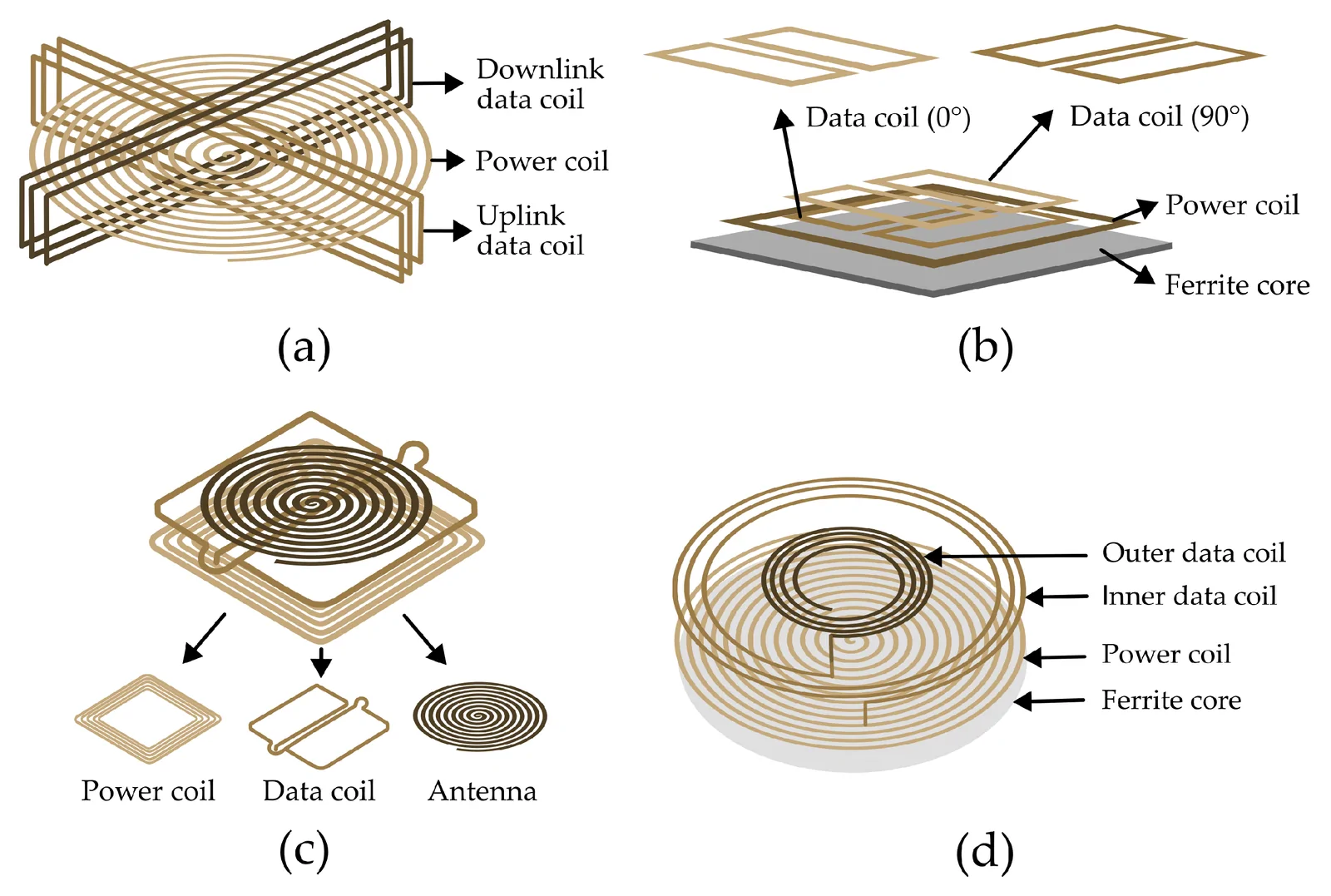
Overview of different cross-coupling mitigation techniques: (**a**) vertical coils, (**b**) figure-eight coils, (**c**) orthogonal DD coils, and (**d**) coaxial figure-eight coils, respectively adapted from [145,146,147,148].

- Four-coil systems: In this approach, two coils are used on the transmitter side and two coils on the receiver side, with one coil in each pair dedicated to power transfer and the other to data communication. Interference may arise because the time-varying magnetic fields generated by the power coils can induce unwanted currents in the data coils. Various techniques have been reported in the literature to mitigate this cross-coupling [13,145]. An overview of these mitigation techniques, reproduced from different publications and combined into a single figure, is given by Figure 18.
  − Time-division multiple access (TDMA): Power and data are transmitted in separate time slots, preventing mutual interference.
  − Vertical coils: Power and data coils are oriented orthogonally to minimize magnetic coupling and induced currents [146].
  − Figure-eight and Double-D coils: The figure-eight and Double-D (DD) data coils are designed such that, in the presence of cross-coupling from the power coil, the induced currents in the two data coil halves are equal in magnitude but opposite in direction [145,147]. As a result, these currents cancel each other, significantly reducing interference from the magnetic field of the power coil. A coaxial version of this 8-figure coil is proposed in [148].
- Hybrid coil-antenna systems: This approach combines inductive coils for power transfer with RF antennas for data communication. Interference is primarily mitigated through frequency separation between the power and data links. When required, additional filtering elements (e.g., wave trappers or trap inductors) and EM shielding can be employed to further suppress coupling between the two links [13]. Nevertheless, complete electromagnetic isolation cannot always be ensured, since conductive antenna structures (e.g., patch antennas) placed in proximity to the inductive coils may support induced eddy currents or distort the surrounding fields.

Overall, multiple wireless links provide greater architectural flexibility, as the power and data channels can be considered separately during system-level optimization, potentially leading to higher efficiency. This approach, however, requires additional hardware, increasing system complexity and challenging implant miniaturization. Furthermore, despite this conceptual separation, both links ultimately need to be physically integrated into a single miniaturized implant, where shared spatial constraints and potential interference inherently couple the two subsystems. Figure 16 can be used to identify suitable WPT and WDT technologies based on the application requirements, as discussed in Section 8.3. Subsequently, the WPT-WDT integration section of Table 6 provides an overview of the available integration architectures described above. Combined, these tools enable the selection of suitable integration strategies for the chosen WPT and WDT technologies, while accounting for key trade-offs related to efficiency, bandwidth, miniaturization, complexity, and cross-coupling.

## 10. Uplink Data Communication

In this work, uplink data communication is defined as the transmission of data from the implant to the external unit. Two types of uplink communication can be distinguished: active and passive telemetry [12]. Active telemetry corresponds to the schemes described above, where the implanted device actively transmits data using modulation techniques (as discussed in Section 8.1) such as ASK, FSK, or PSK. These approaches are typically employed when higher uplink data rates are required, but they come at the cost of increased power consumption in the implant.

In contrast, passive telemetry does not require an active transmitter at the implant. Instead, data are conveyed by modulating the same magnetic or EM field that provides the downlink power (and optionally data) to the implant. When coil-based links are used, this is commonly achieved through LSK, as described in Section 8.1 [12].

For antenna-based systems, a similar principle applies in the form of backscatter communication [12]. In this case, the impedance of the receiving antenna is modulated, causing controlled variations in the reflected EM wave. These reflections are detected by the external transmitter and are decoded as uplink data. This approach allows ultra-low-power communication since the implant does not need to actively generate a signal, only to modulate the reflection. The efficiency of the uplink depends on the contrast between the modulated and unmodulated impedance states, as well as the alignment and separation between the implant and external antenna. Consequently, backscatter communication is particularly suitable for small implantable devices where power consumption and size are critical constraints.

## 11. Electromagnetic Simulation Tools

Several EM simulation tools are commonly used to analyze wireless power and data transfer in implantable applications. These tools are largely based on numerical methods such as the finite element analysis (FEM) or finite integration techniques, enabling full-wave electromagnetic and multiphysics simulations in complex geometries. They are typically employed to evaluate field distributions, tissue absorption, and overall system performance. Popular simulation environments include specialized multiphysics platforms such as Sim4Life^®^ [149], CST Microwave Studio^®^ [150], Ansys HFSS^®^ [151] (optionally coupled with the Ansys OptiSLang optimization framework [152]), and COMSOL Multiphysics^®^ [153].

To accurately represent in vivo operating conditions, simulations are typically performed using anatomically realistic models containing multiple tissue layers and their corresponding electrical and thermal properties. Many commercial software packages provide integrated human body and head models for this purpose. In addition, open-source databases are available, such as those provided by the IT’IS Foundation. This database provides on one hand open-source human body models for different genders, ages, pregnancy-stages, … [154] but also specified head models, like the IXI Head Models [155]. The IT’IS database further provides frequency-dependent dielectric and thermal tissue properties, enabling the development of customized anatomical models for realistic electromagnetic and thermal simulations [156].

Simulations are used throughout the design process to evaluate key wireless-link characteristics such as PTE, coupling coefficient and bandwidth. By systematically exploring the design space, designers can identify the trade-offs between transmission efficiency, implant miniaturization, communication bandwidth, operating distance, and regulatory constraints. As a result, simulation-driven optimization enables rapid virtual prototyping and significantly reduces the number of costly fabrication and measurement iterations required during development. Nevertheless, the final wireless-link performance must ultimately be validated experimentally using physical prototypes. Beyond performance optimization, electromagnetic simulations are indispensable for evaluating compliance with safety requirements. They provide access to quantities that are difficult or even impossible to measure accurately in vivo, including electric- and magnetic-field distributions, localized SAR, Maximum Permissible Exposure (MPE), induced current density, and tissue temperature rise. Consequently, simulation-based assessments have become an essential component of demonstrating compliance with the electromagnetic exposure limits and safety standards discussed in Section 3.

Importantly, safety verification should not be restricted to nominal operating conditions. Once the wireless architecture and optimized design have been selected, compliance is typically assessed under worst-case operating scenarios, including transmitter–receiver misalignment, angular rotation and variations in implantation depth. For example, an inductive link may first be optimized for maximum PTE under ideal alignment conditions. Subsequently, electromagnetic and thermal simulations are repeated for the expected worst-case misalignment scenario. The resulting induced electric field, magnetic field, localized SAR, and tissue temperature rise are then compared with the applicable exposure limits. If any metric exceeds the prescribed limits, parameters such as coil geometry, operating frequency, shielding strategy, or transmit power must be modified and the evaluation repeated. This iterative design process ensures that wireless-link performance and patient safety requirements are satisfied simultaneously. For antenna-based links, the same safety metrics are evaluated and compared against the applicable exposure limits. In addition, because propagating electric fields experience significantly stronger attenuation in biological tissue than the magnetic fields used in inductive links, electromagnetic simulations should also verify sufficient received signal strength and communication performance.

## 12. Conclusions

Inductive coupling remains the most viable approach for WPT in implantable medical devices with the assessed power requirements, as it provides high efficiency over short distances. However, continued device miniaturization inherently degrades magnetic coupling and PTE. While higher operating frequencies and transmit power could theoretically compensate for this loss, practical implementations are fundamentally constrained by safety regulations, including SAR and thermal exposure limits. For WDT, coil-based links are intrinsically bandwidth-limited, whereas RF antenna-based solutions support higher data rates at the cost of increased tissue attenuation. Although carrier frequencies in the GHz range enable larger bandwidths and reduced antenna dimensions, tissue absorption rises rapidly with frequency, effectively restricting practical short-range implantable links to approximately 1 GHz to 10 GHz, for the applications focused on in this paper.

This paper has shown that smart coil design, like optimized geometries, increased conductor thickness, and the strategic manipulation of turn spacing, can significantly reduce proximity losses and skin effects, and increase power transfer efficiency, even under severe miniaturization constraints. The use of ferrite materials was also identified as an effective technique to enhance magnetic coupling and field confinement, thereby contributing to improved efficiency. For antenna-based data links, a wide range of miniaturization techniques, such as meandered lines to lengthen the current path, high-permittivity substrates, and stacked or folded geometries, were reviewed, along with additional strategies like impedance loading and parasitic element integration to optimize both power transfer efficiency and bandwidth. Impedance matching, for example using π- or T-network topologies, is essential for minimizing reflection losses and ensuring stable operation in lossy biological tissue with frequency-dependent dielectric properties. Additionally, modulation schemes such as OOK and FSK were identified as the most robust and energy-efficient. While FSK provides improved noise resilience and supports reliable high-speed data transmission, OOK is often sufficient and preferred due to its simplicity.

At the system level, architectural strategies such as multi-coil configurations, active rectifier circuits, and high-efficiency Class-E transmitter topologies were shown to significantly enhance overall link performance, particularly under conditions of weak coupling and stringent size constraints. The integration of WPT and WDT was evaluated using both single-link and multi-link architectures. While single-link solutions offer compactness and reduced hardware complexity, they impose inherent trade-offs between power transfer efficiency and data bandwidth due to shared channel constraints. In contrast, multi-link architectures provide greater design flexibility and performance margins, especially when interference mitigation techniques such as orthogonal coil placement or frequency separation are employed, albeit at the cost of increased system size and complexity. Additionally, uplink communication strategies were examined, highlighting the suitability of passive telemetry methods, including load-shift keying and backscatter communication, for ultralow-power and typically low data rate communication. To support design validation and safety assessment, this work further surveyed electromagnetic simulation tools, including open-source platforms incorporating anatomical models and frequency-dependent tissue properties.

By combining key design parameters, regulatory constraints, and integration strategies, this paper provides a clear and practical foundation for designing safe, high-performance miniaturized wireless links. To support such designs, Table 6 provides an overview of the key techniques for optimized WPT and WDT links, covering coil-, antenna-, circuit-, and system-level strategies. It also provides a qualitative comparison of single- and multi-link WPT/WDT integration architectures, highlighting the associated trade-offs in bandwidth, efficiency, miniaturization, complexity, and cross-coupling. Combined with the technology selection flowchart in Figure 16, readers can first identify suitable wireless power and data transfer technologies based on application requirements and subsequently consult Table 6 to refine the design and select the most appropriate integration architecture.

**Table 6:** Concluding design guidelines for optimized WPT and WDT links in miniaturized high-performance implants.

**WPT: Coils**				**WDT: Coils**		
**PTE**	**Misalignment**		**Size**	**BW**		
*Section 7*	*Section 7*		*Section 7*	*Section 8.1*		
↑ *k* (reduce misalignment) ↑ *Q* (reduce $R_{\mathrm{dc}}$ and $R_{\mathrm{ac}}$) Ferrites SP/LCC (multi-coil) topology Class-E PA + low-drop rectifier Impedance matching	Coaxial alignment Magnets Anatomy-constrained Multi-coil topology Verify worst-case offset		f↑ (smaller coils) Multi-layer coil Optimize turns, spacing, wire ∅ Trade-off *R* vs. *L*	${\mathrm{BW}}_{\mathrm{req}}\Rightarrow Q_{\max}\approx \frac{f_{0}}{{\mathrm{BW}}_{\mathrm{req}}}$ De-Qing (for TDMA) OOK/FSK for downlink LSK for uplink Reuse coil power		
**WDT: Antennas**						
**BW + gain**	**Miniaturization**	**Optimal freq.**	**Antenna type**	**Gain enhancement**	**BW enhancement**	
*Section 8.2*	*Section 8.2.4*	*Section 8.2.1*	*Section 8.2.2*	*Section 8.2.6*	*Section 8.2.7*	
Separate RF link for ↑ bit/s Match antenna impedance Trade-off *Q* vs. BW Max achiev. BW: Equation (12) Max achiev. gain: Equation (23)	High-$\varepsilon_{r}$ substrate Meander or fractal path PIFA/shorting pin SRR/DGS loading Stacking or folding	Balance $e_{\mathrm{NF}}$ and $e_{\mathrm{prop}.}$ Ensure δ=1/α≥d Practical range: 1–10 GHz	PCB: recommended Flexible antenna Spiral antenna Helical antenna ME antenna	Superstrate (high-$\varepsilon_{r}$) Capsule/air gap: ↑ size Encapsulation layer Dielectric capsule filling Impedance matching	Loop radiators Meandered DGS Parasitic patches Multiband operation MIMO antennas	
**WPT/WDT Integration** * Section 9*						
**Technique**	**WPT-WDT (link type)**	**Seperation Method**	**PTE-BW trade-off**	**Miniaturization**	**Cross-coupling**	**Complexity**
Power-carrier data modulation	Coil–Coil (single)	Protocol	Strong	Excellent	None	Low
Power/Data TDMA de-*Q*ing	Coil–Coil (single)	Time	None	Excellent	None	Moderate
Coil reused as antenna	Coil-Antenna (single)	Freq.	Moderate	Very good	Filtering required	Moderate
TDMA Four-coil system	Coil–Coil (multi)	Time & physical	None	Moderate	None	High
2 pairs of vertical coils	Coil–Coil (multi)	Physical	None	Difficult	very Low	High
2 pairs of double-D coils	Coil–Coil (multi)	Physical	None	Moderate	Very low	High
Hybrid coil–antenna	Coil–Antenna (multi)	Freq.	None	Difficult	Low (freq. separation)	High
**Final design directions:** Use resonant inductive coupling for efficient WPT. Use the same coil link for WDT only when compactness and simplicity dominate over data rate. Use a separate antenna-based RF link when high data rate is required. In all cases, the final design must be validated using EM and thermal simulations, including PTE, BW, link margin, SAR, MPE, temperature rise, tissue detuning and worst-case misalignment.						

Future research should focus on identifying the fundamental performance limits, isolating the most critical technological bottlenecks, and developing novel solutions to overcome these challenges. Addressing these aspects is essential to enable the next generation of implantable devices that are smaller, smarter, and safer. 

## Figures and Tables

**Table 1:** Regulatory authorities for implantable wireless medical devices.

Region	Medical Device	Radio/Spectrum
United States	FDA [27]	FCC [28]
European Union	MDR [29]	RED [30]
Canada	Health Canada [31]	ISED [32,33]
China	NMPA [34]	MIIT/SRRC [35]
Japan	PMDA [36]	MIC [37]
Australia	TGA [38]	ACMA [39]
New Zealand	Medsafe [40]	RSM [41]

**Table 2:** Approximate tissue attenuation of wireless power transfer mechanisms, together with the typical receiver size for usable PTE and representative data rates reported for implantable applications.

Mechanism	Carrier	Tissue Attenuation		Typical RX Size for Usable PTE	Data Rate
		Freq.	Attenuation		
Inductive	Mag. field	21 MHz	0.066 dB/cm [61]	0.5–$5\;{\mathrm{cm}}^{2}$ [6]	≲5 Mbps [10]
Radiative	EM wave	403 MHz	0.834 dB/cm [62]	0.5–$2\;{\mathrm{cm}}^{2}$ [6]	≳100 Mbps [10]
		916 MHz	1 dB/cm [62]		
		2.4 GHz	1.89 dB/cm [62]		
Optical	Light wave	450– 700 nm	>170 dB/cm [61]	≤$0.01\;{\mathrm{cm}}^{2}$ [6]	≲5 Mbps [10]
Acoustic	Press. wave	1 MHz	0.54 dB/cm [61]	0.05–$0.5\;{\mathrm{cm}}^{2}$ [6]	≲1 Mbps [10]
		1 MHz	0.73 dB/cm [62,63]		
Capacitive	Elec. field	kHz–MHz	≈10 dB/cm [5]	2–$10\;{\mathrm{cm}}^{2}$ [6]	≲1 Mbps [64]

**Table 3:** Qualitative comparison of wireless technologies with respect to application-level metrics.

Technology	WPT	WDT	Shared Constraints		
	PTE	BW	Misalignment sqs	Distance	S
Inductive coupling (coils)	✓	✓	×	×	✓
Resonant inductive coupling	✓✓	✓	✓	✓	✓
RF antennas (GHz)	×	✓✓	✓	✓	✓
Optical links	××	✓✓	×	×	✓
Acoustic (ultrasound)	✓	×	×	✓✓	✓
Capacitive coupling	××	✓	✓	××	××

**Table 4:** Maximum $f_{\max}$ and optimum $f_{\mathrm{opt}}$ operating frequencies as a function of TX–RX separation, together with the corresponding tissue attenuation constant α.

$\Delta_{\mathrm{TX-RX}}$ [mm]	$f_{\max,\;\mathrm{only}\;\mathrm{prop}}$ [GHz]	$f_{\mathrm{opt},\;\mathrm{TM}+\mathrm{prop}}$ [GHz]	$f_{\mathrm{opt},\;\mathrm{TE}+\mathrm{prop}}$ [Np/m]	$\alpha (f_{\mathrm{opt},\;\mathrm{TM}+\mathrm{prop}})$ [Np/m]	$\alpha (f_{\mathrm{opt},\;\mathrm{TE}+\mathrm{prop}})$
2	15.04	11.66	4.421	333.142	81.492
5	8.25	7.522	4.421	172.372	81.4918
10	5.079	4.524	4.320	84.1809	79.96
20	1.970	4.421	1.715	81.49	44.98
30	1.055	4.320	0.819	79.968	28.36

## Data Availability

No new data were created or analyzed in this study. Data sharing is not applicable to this article.

## References

[1] Center for Devices and Radiological Health (2021). Benefits and Risks of Cochlear Implants. https://www.fda.gov/medical-devices/cochlear-implants/benefits-and-risks-cochlear-implants.

[2] Yoo B., Kim J., Kim M., Kim M., Choi J., Kim D., Park G., Park S.-Y. (2026). A 6.78-MHz Wireless, Mode-Convertible Single-Stage Resonant CC/CV Battery Charger for Implantable Biomedical Devices. IEEE Trans. Biomed. Circuits Syst..

[3] Zhu H., Tahir M., Wu X., Hu S. (2024). High-Performance Wireless Power and Data Transmission System for Medical Implant Devices Using ASK Modulation. Energies.

[4] Kaim V., Kanaujia B.K., Kumar S., Choi H.C., Kim K.W., Rambabu K. (2022). Electrically Small Circularly Polarized UWB Intraocular Antenna System for Retinal Prosthesis. IEEE Trans. Biomed. Eng..

[5] Barbruni G.L., Ros P.M., Demarchi D., Carrara S., Ghezzi D. (2020). Miniaturised Wireless Power Transfer Systems for Neurostimulation: A Review. IEEE Trans. Biomed. Circuits Syst..

[6] Khan S.R., Pavuluri S.K., Cummins G., Desmulliez M.P.Y. (2020). Wireless Power Transfer Techniques for Implantable Medical Devices: A Review. Sensors.

[7] Van Mulders J., Delabie D., Lecluyse C., Buyle C., Callebaut G., Van Der Perre L., De Strycker L. (2022). Wireless Power Transfer: Systems, Circuits, Standards, and Use Cases. Sensors.

[8] Aliqab K., Nadeem I., Khan S.R. (2023). A Comprehensive Review of In-Body Biomedical Antennas: Design, Challenges and Applications. Micromachines.

[9] Benaissa M., Chaabane A., Attia H., Al-Naib I. (2025). Innovations and Challenges in RF Antenna Technologies for Implantable Medical Devices Communication. IEEE J. Microw..

[10] Lin C., Zhu S., Han C., Yu S., Zhang Z., Mao J. (2024). A Review of Wireless Intra-Body Communication for Neural Implants. Neuroelectronics.

[11] Essa A., Almajali E., Mahmoud S., Amaya R.E., Alja’Afreh S.S., Ikram M. (2024). Wireless Power Transfer for Implantable Medical Devices: Impact of Implantable Antennas on Energy Harvesting. IEEE Open J. Antennas Propag..

[12] Karimi M.J., Schmid A., Dehollain C. (2021). Wireless Power and Data Transmission for Implanted Devices via Inductive Links: A Systematic Review. IEEE Sens. J..

[13] Yao Y., Sun P., Liu X., Wang Y., Xu D. (2022). Simultaneous Wireless Power and Data Transfer: A Comprehensive Review. IEEE Trans. Power Electron..

[14] Hong B., Wei X., Shi C., Mai S., Tang X., Wang Z. (2026). A Review of Wireless Power and Data Transfer Systems for Biomedical Implants. Neuroelectronics.

[15] Cochlear How Do Cochlear Hearing Implants Work?. https://www.cochlear.com/us/en/home/diagnosis-and-treatment/how-cochlear-solutions-work/cochlear-implants/how-cochlear-implants-work.

[16] About Cochlear Implants. https://www.cochlear.com/us/en/home/diagnosis-and-treatment/how-cochlear-solutions-work/cochlear-implants.

[17] Boeckx S., Polfliet P., De Strycker L., Van Der Perre L. (2025). Computationally Efficient Impact Estimation of Coil Misalignment for Magnet-Free Cochlear Implants. Sensors.

[18] Deep N., Dowling E., Jethanamest D., Carlson M. (2018). Cochlear Implantation: An Overview. J. Neurol. Surg. Part B Skull Base.

[19] Wikimedia Commons Contributors (2022). Advanced Bionics Cochlear Implant Processor (Naïda). https://commons.wikimedia.org/wiki/File:Advanced_Bionics_cochlear_implant_processor,_brand-named_%22Na%C3%ADda%22_(third-generation_behind-the-ear_processor).jpg.

[20] Wikimedia Commons (2009). File:Cochearimplants.JPG. https://commons.wikimedia.org/wiki/File:Cochearimplants.JPG.

[21] Wikimedia Commons (2008). File:Cochleaimplantat.jpg. https://commons.wikimedia.org/wiki/File:Cochleaimplantat.jpg.

[22] ElAassar A., Foad Y., El-Anwar M. (2015). Non-mastoidectomy Cochlear Implant Approaches: A Literature review. Int. Arch. Otorhinolaryngol..

[23] Yoon Y.H., Yue L., Humayun M.S. (2020). Argus II Prosthetic Vision.

[24] Wikimedia Commons (2025). File:Schematic Diagram of the Eye and Retina.svg. https://commons.wikimedia.org/wiki/File:Schematic_diagram_of_the_eye_and_retina.svg.

[25] Wikimedia Commons (2021). File:Ray-Ban Stories.jpg. https://commons.wikimedia.org/wiki/File:Ray-Ban_Stories.jpg.

[26] Stanford Artificial Retina Project. https://med.stanford.edu/artificial-retina.html.

[27] U.S. Food and Drug Administration. https://www.fda.gov/.

[28] Federal Communications Commission https://www.fcc.gov/.

[29] Medical Device Regulation. https://www.medical-device-regulation.eu/.

[30] European Commission Radio Equipment Directive (RED). https://single-market-economy.ec.europa.eu/sectors/electrical-and-electronic-engineering-industries-eei/radio-equipment-directive-red_en.

[31] Legal Services Branch, Health Canada (2026). Consolidated Federal Laws of Canada, Medical Devices Regulations. https://laws-lois.justice.gc.ca/eng/regulations/sor-98-282/.

[32] ISED Canada (2026). Innovation, Science and Economic Development Canada Official Site. https://ised-isde.canada.ca/site/ised/en.

[33] Spectrum and Telecommunications Sector (STS) (2021). RSS-Gen—General Requirements for Compliance of Radio Apparatus. https://ised-isde.canada.ca/site/spectrum-management-telecommunications/en/devices-and-equipment/radio-equipment-standards/radio-standards-specifications-rss/rss-gen-general-requirements-compliance-radio-apparatus.

[34] National Medical Products Administration. https://english.nmpa.gov.cn/.

[35] MPR China Certification (2025). SRRC Certification for China. https://www.china-certification.com/en/china-srrc-type-approval/.

[36] Pharmaceuticals and Medical Devices Agency. https://www.pmda.go.jp/english/.

[37] Ministry of Internal Affairs and Communications. https://www.soumu.go.jp/english/.

[38] Therapeutic Goods Administration. https://www.tga.gov.au/.

[39] Australian Communications and Media Authority. https://www.acma.gov.au/.

[40] MedSafe Home Page. https://www.medsafe.govt.nz/.

[41] (2026). Radio Spectrum Management New Zealand. https://www.rsm.govt.nz/.

[42] (2016). Medical Devices—Quality Management Systems—Requirements for Regulatory Purposes.

[43] (2019). Medical Devices—Application of Risk Management to Medical Devices.

[44] (2018). Biological Evaluation of Medical Devices.

[45] (2014). Implants for Surgery—Active Implantable Medical Devices.

[46] (2019). Implants for Surgery—Active Implantable Medical Devices—Part 7: Particular Requirements for Cochlear and Auditory Brainstem Implant Systems.

[47] (2015). Implants for Surgery—Active Implantable Medical Devices—Part 1: General Requirements for Safety, Marking and for Information to Be Provided by the Manufacturer.

[48] (2016). Short Range Devices (SRD).

[49] ECO Documentation. https://docdb.cept.org.

[50] International Commission on Non-Ionizing Radiation Protection AIM, Status & History. https://www.icnirp.org/en/about-icnirp/aim-status-history/index.html.

[51] IEEE Standards Association https://standards.ieee.org/.

[52] Health Canada (2015). Limits of Human Exposure to Radiofrequency Electromagnetic Energy in the Frequency Range from 3 kHz to 300 GHz.

[53] Mohan A., Kumar N. (2024). Implantable antennas for biomedical applications: A systematic review. BioMed. Eng. OnLine.

[54] Liu C., Guo Y.X., Sun H., Xiao S. (2014). Design and Safety Considerations of an Implantable Rectenna for Far-Field Wireless Power Transfer. IEEE Trans. Antennas Propag..

[55] Publications Office of the European Union (1999). CELEX1, 1999/519/EC: Council Recommendation of 12 July 1999 on the Limitation of Exposure of the General Public to Electromagnetic Fields (0 Hz to 300 GHz).

[56] Federal Communications Commission (2024). 47 CFR §1.1310—Radiofrequency Radiation Exposure Limits.

[57] 47 CFR Part 18 Subpart C—Technical Standards. https://www.ecfr.gov/current/title-47/chapter-I/subchapter-A/part-18/subpart-C?toc=1.

[58] (2024). Industrial, Scientific and Medical Equipment—Radio-Frequency Disturbance Characteristics—Limits and Methods of Measurement.

[59] Van Rhoon G.C., Samaras T., Yarmolenko P.S., Dewhirst M.W., Neufeld E., Kuster N. (2013). CEM43 °C thermal dose thresholds: A potential guide for magnetic resonance radiofrequency exposure levels?. Eur. Radiol..

[60] Whalen A.J., Fried S.I. (2023). Thermal safety considerations for implantable micro-coil design. J. Neural Eng..

[61] Van Helleputte N., Even A.J.G., Leonardi F., Stanzione S., Song M., Garripoli C., Sijbers W., Liu Y.H., Van Hoof C. (2020). Miniaturized Electronic Circuit Design Challenges for Ingestible Devices. J. Microelectromech. Syst..

[62] Dove I. (2014). Analysis of Radio Propagation Inside the Human Body for In-Body Localization Purposes. Master’s Thesis.

[63] Quarato C.M.I., Lacedonia D., Salvemini M., Tuccari G., Mastrodonato G., Villani R., Fiore L.A., Scioscia G., Mirijello A., Saponara A. (2023). A Review on Biological Effects of Ultrasounds: Key Messages for Clinicians. Diagnostics.

[64] Narayanamoorthi R. (2019). Modeling of Capacitive Resonant Wireless Power and Data Transfer to Deep Biomedical Implants. IEEE Trans. Compon. Packag. Manuf. Technol..

[65] Boeckx S., Polfliet P., De Strycker L., Van der Perre L. (2026). Electric Field Attenuation Techniques for Inductive Wireless Charging of Medical Implants. arXiv.

[66] Agu D.U., Leece M., Alcala-Medel J., Sahdev A., Lim J., Olsen M., Ngan B., Kim Y., Li Y. (2020). Investigation of Dominant Wave Mechanisms and Optimal Antenna Excitation for Body-Centric Wireless Propagation. Prog. Electromagn. Res. C.

[67] Ulaby F.T., Michielssen E., Ravaioli U. (2015). Fundamentals of Applied Electromagnetics.

[68] Salchak Y.A., Albadri N.M., Bjorkman T., Lau C., Nadimi E.S., Bollen P., Espinosa H.G., Thiel D.V. (2022). In-vivo Experimental Validation of the Attenuation Path Loss Model for Localization of Wireless Implanted Transmitters at 2.45 GHz. IEEE Access.

[69] (2024). Cochlear Implant Electronic Device Provides Sound for Deaf. https://lmhofmeyr.co.za/conditions/conditions-we-specialise-in/cochlear-implant/.

[70] Van Schuylenbergh K., Puers R. (2009). Inductive Powering: Basic Theory and Application to Biomedical Systems.

[71] Chu L.J. (1948). Physical Limitations of Omni-Directional Antennas. J. Appl. Phys..

[72] Balanis C.A. (2016). Antenna Theory: Analysis and Design.

[73] Pozar D.M. (2012). Microwave Engineering.

[74] Jasim M., Al-Gburi A.J.A., Hanif M., Dayo Z.A., Ismail M.M., Zakaria Z. (2024). An extensive review on implantable antennas for biomedical applications: Health considerations, geometries, fabrication techniques, and challenges. Alex. Eng. J..

[75] Soliman M.M., Chowdhury M.E.H., Khandakar A., Islam M.T., Qiblawey Y., Musharavati F., Nezhad E.Z. (2021). Review on Medical Implantable Antenna Technology and Imminent Research Challenges. Sensors.

[76] Giancoli D.C. (2014). Physics: Principles with Applications.

[77] Hussain I., Woo D.K. (2022). Inductance Calculation of Single-Layer Planar Spiral Coil. Electronics.

[78] Severns R. (2019). Coil Length-to-Diameter Ratios for Maximum Q in LFMF Antenna Loading Inductors; Technical Report. https://antennasbyn6lf.com/Misc_notes/Optimum%20Inductor%20l-D%20ratios.pdf.

[79] Awuah C.M., Danuor P., Moon J.I., Jung Y.B. (2023). Novel coil design and analysis for high-power wireless power transfer with enhanced Q-factor. Sci. Rep..

[80] Sautter M., Sautter N., Shellock F.G. (2022). Near field communication (NFC) device: Evaluation of MRI issues. Magn. Reson. Imaging.

[81] Dewey R.S., Bowtell R., Kitterick P. (2022). A global survey of healthcare professionals undertaking MRI of patients with cochlear implants: A heterogeneity of practice and opinions. Br. J. Radiol..

[82] Gubbels S.P., McMenomey S.O. (2006). Safety Study of the Cochlear Nucleus^®^ 24 Device with Internal Magnet in the 1.5 Tesla Magnetic Resonance Imaging Scanner. Laryngoscope.

[83] Dewey R.S., Kitterick P.T. (2021). Cochlear implant user perceptions of magnetic resonance imaging. Cochlear Implant. Int..

[84] Mahmood A.I., Gharghan S.K., Eldosoky M.A., Soliman A.M. (2022). Near-field wireless power transfer used in biomedical implants: A comprehensive review. IET Power Electron..

[85] Sohn Y.H., Choi B.H., Lee E.S., Lim G.C., Cho G., Rim C.T. (2015). General Unified Analyses of Two-Capacitor Inductive Power Transfer Systems: Equivalence of Current-Source SS and SP Compensations. IEEE Trans. Power Electron..

[86] Shevchenko V., Husev O., Strzelecki R., Pakhaliuk B., Poliakov N., Strzelecka N. (2019). Compensation Topologies in IPT Systems: Standards, Requirements, Classification, Analysis, Comparison and Application. IEEE Access.

[87] Tran M.T., Thekkan S., Polat H., Tran D.D., Baghdadi M.E., Hegazy O. (2023). Inductive Wireless Power Transfer Systems for Low-Voltage and High-Current Electric Mobility Applications: Review and Design Example. Energies.

[88] Triviño Cabrera A., Sánchez J.A.A. (2018). A Review on the Fundamentals and Practical Implementation Details of Strongly Coupled Magnetic Resonant Technology for Wireless Power Transfer. Energies.

[89] Rozman M., Fernando M., Adebisi B., Rabie K., Kharel R., Ikpehai A., Gacanin H. (2017). Combined Conformal Strongly-Coupled Magnetic Resonance for Efficient Wireless Power Transfer. Energies.

[90] Basir A., Yoo H. (2020). Efficient Wireless Power Transfer System With a Miniaturized Quad-Band Implantable Antenna for Deep-Body Multitasking Implants. IEEE Trans. Microw. Theory Tech..

[91] Ziemer R.E., Tranter W.H. (2015). Principles of Communications: Systems, Modulation, and Noise.

[92] Wheeler H. (1947). Fundamental Limitations of Small Antennas. Proc. IRE.

[93] Capozzoli A., Curcio C., D’Agostino F., Liseno A. (2024). A Review of the Antenna Field Regions. Electronics.

[94] Gao M., Sipus Z., Skrivervik A.K. (2023). Analytic Approximation of In-Body Path Loss for Implanted Antennas. IEEE Open J. Antennas Propag..

[95] Malik N.A., Sant P., Ajmal T., Ur-Rehman M. (2020). Implantable Antennas for Bio-Medical Applications. IEEE J. Electromagn. RF Microw. Med. Biol..

[96] Xu L.J., Bo Y., Lu W.J., Zhu L., Guo C. (2019). Circularly Polarized Annular Ring Antenna With Wide Axial-Ratio Bandwidth for Biomedical Applications. IEEE Access.

[97] Shah S.A.A., Yoo H. (2018). Scalp-Implantable Antenna Systems for Intracranial Pressure Monitoring. IEEE Trans. Antennas Propag..

[98] Das S., Mitra D. (2018). A compact wideband flexible implantable slot antenna design with enhanced gain. IEEE Trans. Antennas Propag..

[99] Fu Y., Lei J., Zou X., Guo J. (2019). Flexible antenna design on PDMS substrate for implantable bioelectronics applications. Electrophoresis.

[100] Lee S.H., Chang K., Kim K.J., Yoon Y.J. A conical spiral antenna for wideband capsule endoscope system. Proceedings of the 2008 IEEE Antennas and Propagation Society International Symposium.

[101] Wang X., Shi J., Xu L., Wang J. A Wideband Miniaturized Implantable Antenna for Biomedical Application at HBC Band. Proceedings of the 2018 Cross Strait Quad-Regional Radio Science and Wireless Technology Conference (CSQRWC).

[102] Zainud-Deen S.H., Malhat H.A.E.A., Balabel A.A. (2019). Octafilar helical antenna for circular polarization wireless capsule endoscopy applications. Wirel. Pers. Commun..

[103] Shah I.A., Zada M., Yoo H. (2019). Design and Analysis of a Compact-Sized Multiband Spiral-Shaped Implantable Antenna for Scalp Implantable and Leadless Pacemaker Systems. IEEE Trans. Antennas Propag..

[104] Samanta G., Mitra D. (2019). Dual-Band circular polarized flexible implantable antenna using reactive impedance substrate. IEEE Trans. Antennas Propag..

[105] Omran M., Ghobadi C., Nourinia J., Shokri M. (2024). Miniaturized Ss-Shaped CP Circular Patch Antenna Design for Implantable Medical Device Applications. Int. J. RF Microw. Comput.-Aided Eng..

[106] Nan T., Lin H., Gao Y., Matyushov A., Yu G., Chen H., Sun N., Wei S., Wang Z., Li M. (2017). Acoustically actuated ultra-compact NEMS magnetoelectric antennas. Nat. Commun..

[107] Mazón-Maldonado L., Zhang J., Cerezo-Sánchez M., Baghini M.S., Parvizi R., Heidari H. (2025). MEMS-Based Magnetoelectric Antennas for Wireless Power Transmission in Brain-Implantable Devices. Adv. Mater. Technol..

[108] Das D., Nasrollahpour M., Xu Z., Zaeimbashi M., Martos-Repath I., Mittal A., Khalifa A., Cash S.S., Shrivastava A., Sun N.X. (2020). A Radio Frequency Magnetoelectric Antenna Prototyping Platform for Neural Activity Monitoring Devices with Sensing and Energy Harvesting Capabilities. Electronics.

[109] Satitchantrakul T., Siponkoski T., Nelo M., Bai Y., Soh P.J. (2026). Magnetoelectric antennas: Fundamentals, state-of-the-art, challenges, and future perspectives. APL Electron. Devices.

[110] Liang X., Chen H., Sun N., Lin H., Sun N.X. Novel Acoustically Actuated Magnetoelectric Antennas. Proceedings of the 2018 IEEE International Symposium on Antennas and Propagation & USNC/URSI National Radio Science Meeting.

[111] Ma Y., Liu C., Huang Y., Ke H., Liu X. (2025). Combined Magnetoelectric/Coil Receiving Antenna for Biomedical Wireless Power Transfer. IEEE J. Electromagn. RF Microw. Med. Biol..

[112] Chen H. (2021). Integrated RF Devices Based on Magnetoelectric Coupling. Ph.D. Thesis.

[113] Cai Y., Joy B.C., Desbiolles B.X.E., Schell V., Yadav S., Bono D.C., Sarkar D. (2025). Low-Frequency Sub-0.5 mm Magnetoelectric Antenna for Wireless Power Harvesting in Injectable Deep-Tissue Implants. IEEE Trans. Antennas Propag..

[114] Liu C., Guo Y.X., Xiao S. (2014). Capacitively Loaded Circularly Polarized Implantable Patch Antenna for ISM Band Biomedical Applications. IEEE Trans. Antennas Propag..

[115] Arar S. (2023). Using the Smith Chart to Design a T and PI Matching Network.

[116] Xu L.J., Guo Y.X., Wu W. (2014). Miniaturized Dual-Band Antenna for Implantable Wireless Communications. IEEE Antennas Wirel. Propag. Lett..

[117] Liu C., Guo Y.X., Xiao S. (2016). A Review of Implantable Antennas for Wireless Biomedical Devices. Forum Electromagn. Res. Methods Appl. Technol. (FERMAT).

[118] Sulaiman N.H., Samsuri N.A., Rahim M.K.A., Seman F.C., Inam M. (2018). Compact Meander Line Telemetry Antenna for Implantable Pacemaker Applications. Indones. J. Electr. Eng. Comput. Sci..

[119] Li R., Guo Y.X., Du G. (2018). A Conformal Circularly Polarized Antenna for Wireless Capsule Endoscope Systems. IEEE Trans. Antennas Propag..

[120] Zhang H., Li L., Liu C., Guo Y.X., Wu S. (2017). Miniaturized implantable antenna integrated with split resonate rings for wireless power transfer and data telemetry. Microw. Opt. Technol. Lett..

[121] Duan Z., Guo Y.X., Xue R.F., Je M., Kwong D.L. (2012). Differentially Fed Dual-Band Implantable Antenna for Biomedical Applications. IEEE Trans. Antennas Propag..

[122] Liu N.C., Guo N.Y.X., Xiao N.S. (2012). Compact Dual-Band Antenna for Implantable Devices. IEEE Antennas Wirel. Propag. Lett..

[123] Duan Z., Guo Y.X., Je M., Kwong D.L. (2014). Design and in Vitro Test of a Differentially Fed Dual-Band Implantable Antenna Operating at MICS and ISM Bands. IEEE Trans. Antennas Propag..

[124] Li H., Guo Y.X., Liu C., Xiao S., Li L. (2015). A Miniature-Implantable Antenna for MedRadio-Band Biomedical Telemetry. IEEE Antennas Wirel. Propag. Lett..

[125] Xu L.J., Guo Y.X., Wu W. (2015). Miniaturized Circularly Polarized Loop Antenna for Biomedical Applications. IEEE Trans. Antennas Propag..

[126] Liu C., Guo Y.X., Xiao S. (2014). Circularly Polarized Helical Antenna for ISM-Band Ingestible Capsule Endoscope Systems. IEEE Trans. Antennas Propag..

[127] Lee C.m., Yo T.c., Luo C.h., Tu C.h., Juang Y.z. (2007). Compact broadband stacked implantable antenna for biotelemetry with medical devices. Electron. Lett..

[128] Karacolak T., Hood A., Topsakal E. (2008). Design of a Dual-Band Implantable Antenna and Development of Skin Mimicking Gels for Continuous Glucose Monitoring. IEEE Trans. Microw. Theory Tech..

[129] Liu W.C., Chen S.H., Wu C.M. (2009). Bandwidth enhancement and size reduction of an implantable PIFA antenna for biotelemetry devices. Microw. Opt. Technol. Lett..

[130] Kiourti A., Nikita K.S. (2012). Miniature Scalp-Implantable Antennas for Telemetry in the MICS and ISM Bands: Design, Safety Considerations and Link Budget Analysis. IEEE Trans. Antennas Propag..

[131] Huang F.J., Lee C.M., Chang C.L., Chen L.K., Yo T.C., Luo C.H. (2011). Rectenna Application of Miniaturized Implantable Antenna Design for Triple-Band Biotelemetry Communication. IEEE Trans. Antennas Propag..

[132] Mahalakshmi N., Thenmozhi A. (2017). Design of hexagon shape bow-tie patch antenna for implantable bio-medical applications. Alex. Eng. J..

[133] Kumar S.A., Shanmuganantham T. (2013). Coplanar waveguide-fed ISM band implantable crossed-type triangular slot antenna for biomedical applications. Int. J. Microw. Wirel. Technol..

[134] Chien T.F., Cheng C.M., Yang H.C., Jiang J.W., Luo C.H. (2010). Development of Nonsuperstrate Implantable Low-Profile CPW-Fed Ceramic Antennas. IEEE Antennas Wirel. Propag. Lett..

[135] Yang H.C., Chien T.F., Cheng C.M., Chen K.H. (2010). Develop an Implantable CPW-Fed Antenna on the MgTa_1.5_Nb_0.5_O_6_ Ceramic Substrate. Key Eng. Mater..

[136] Yuliandoko H., Setijadi E., Handayani P. Miniaturization Microstrip Antenna for WPT Implantable Antenna by Using Fractal and DGS Techniques. Proceedings of the 2023 6th International Seminar on Research of Information Technology and Intelligent Systems (ISRITI).

[137] Li R., Li B., Du G., Sun X., Sun H. (2019). A Compact Broadband Antenna with Dual-Resonance for Implantable Devices. Micromachines.

[138] Luo L., Hu B., Wu J., Yan T., Xu L.J. (2019). Compact dual-band antenna with slotted ground for implantable applications. Microw. Opt. Technol. Lett..

[139] Kiourti A., Christopoulou M., Nikita K.S. Performance of a novel miniature antenna implanted in the human head for wireless biotelemetry. Proceedings of the 2011 IEEE International Symposium on Antennas and Propagation (APSURSI).

[140] Sánchez-Fernández C.J., Quevedo-Teruel O., Requena-Carrión J., Inclán-Sánchez L., Rajo-Iglesias E. (2010). Dual-band microstrip patch antenna based on short-circuited ring and spiral resonators for implantable medical devices. IET Microw. Antennas Propag..

[141] Nikolayev D., Zhadobov M., Coq L.L., Karban P., Sauleau R. (2017). Robust Ultraminiature Capsule Antenna for Ingestible and Implantable Applications. IEEE Trans. Antennas Propag..

[142] Dissanayake T., Esselle K.P., Yuce M.R. (2009). Dielectric Loaded Impedance Matching for Wideband Implanted Antennas. IEEE Trans. Microw. Theory Tech..

[143] Krikidis I., Timotheou S., Nikolaou S., Zheng G., Ng D.W.K., Schober R. (2014). Simultaneous wireless information and power transfer in modern communication systems. IEEE Commun. Mag..

[144] Wu J., Zhao C., Lin Z., Du J., Hu Y., He X. (2015). Wireless power and data transfer via a common inductive link using frequency division multiplexing. IEEE Trans. Ind. Electron..

[145] Jow U.M., Ghovanloo M. (2010). Optimization of Data Coils in a Multiband Wireless Link for Neuroprosthetic Implantable Devices. Biomed. Circuits Syst. IEEE Trans..

[146] Ghovanloo M., Atluri S. (2007). A Wide-Band Power-Efficient Inductive Wireless Link for Implantable Microelectronic Devices Using Multiple Carriers. IEEE Trans. Circuits Syst. I Regul. Pap..

[147] Li X., Hu J., Li Y., Wang H., Liu M., Deng P. (2019). A Decoupled Power and Data-Parallel Transmission Method With Four-Quadrant Misalignment Tolerance for Wireless Power Transfer Systems. IEEE Trans. Power Electron..

[148] Rathge C., Kuschner D. High efficient inductive energy and data transmission system with special coil geometry. Proceedings of the European Conference on Power Electronics and Applications.

[149] Sim4Life for Students Free Cloud-Based Simulation Platform for Education and Research. https://sim4life.swiss/students.

[150] Dassault Systèmes (2025). CST Studio Suite 2025. Electromagnetic Simulation Solvers. https://www.3ds.com/products/simulia/cst-studio-suite/electromagnetic-simulation-solvers.

[151] (2025). 3D High Frequency Simulation Software.

[152] (2025). Process Integration and Design Optimization Software.

[153] (2025). COMSOL Multiphysics: Multiphysics Software for Optimizing Designs.

[154] (2015). DUKE.

[155] (2024). IXI HEADS.

[156] Hasgall P.A., Di Gennaro F., Baumgartner C., Neufeld E., Lloyd B., Gosselin M.C., Payne D., Klingenböck A., Kuster N. (2018). IT’IS Database for Thermal and Electromagnetic Parameters of Biological Tissues.

